# An intronic enhancer of *Cebpa* regulates adipocyte differentiation and adipose tissue development via long‐range loop formation

**DOI:** 10.1111/cpr.13552

**Published:** 2023-10-31

**Authors:** Xiaokai Li, Sha Zeng, Li Chen, Yu Zhang, Xuemin Li, Biwei Zhang, Duo Su, Qinjiao Du, Jiaman Zhang, Haoming Wang, Zhining Zhong, Jinwei Zhang, Penghao Li, Anan Jiang, Keren Long, Mingzhou Li, Liangpeng Ge

**Affiliations:** ^1^ State Key Laboratory of Swine and Poultry Breeding Industry Sichuan Agricultural University Chengdu China; ^2^ Livestock and Poultry Multi‐omics Key Laboratory of Ministry of Agriculture and Rural Affairs, College of Animal Science and Technology Sichuan Agricultural University Chengdu China; ^3^ Chongqing Academy of Animal Sciences Chongqing China; ^4^ National Center of Technology Innovation for Pigs Chongqing China; ^5^ Key Laboratory of Pig Industry Science Ministry of Agriculture Chongqing China; ^6^ Jinxin Research Institute for Reproductive Medicine and Genetics Sichuan Jinxin Xi'nan Women's and Children's Hospital Chengdu China

## Abstract

*Cebpa* is a master transcription factor gene for adipogenesis. However, the mechanisms of enhancer–promoter chromatin interactions controlling *Cebpa* transcriptional regulation during adipogenic differentiation remain largely unknown. To reveal how the three‐dimensional structure of *Cebpa* changes during adipogenesis, we generated high‐resolution chromatin interactions of *Cebpa* in 3T3‐L1 preadipocytes and 3T3‐L1 adipocytes using circularized chromosome conformation capture sequencing (4C‐seq). We revealed dramatic changes in chromatin interactions and chromatin status at interaction sites during adipogenic differentiation. Based on this, we identified five active enhancers of *Cebpa* in 3T3‐L1 adipocytes through epigenomic data and luciferase reporter assays. Next, epigenetic repression of Cebpa‐L1‐AD‐En2 or ‐En3 by the dCas9‐KRAB system significantly down‐regulated *Cebpa* expression and inhibited adipocyte differentiation. Furthermore, experimental depletion of cohesin decreased the interaction intensity between Cebpa‐L1‐AD‐En2 and the *Cebpa* promoter and down‐regulated *Cebpa* expression, indicating that long‐range chromatin loop formation was mediated by cohesin. Two transcription factors, RXRA and PPARG, synergistically regulate the activity of Cebpa‐L1‐AD‐En2. To test whether Cebpa‐L1‐AD‐En2 plays a role in adipose tissue development, we injected dCas9‐KRAB‐En2 lentivirus into the inguinal white adipose tissue (iWAT) of mice to suppress the activity of Cebpa‐L1‐AD‐En2. Repression of Cebpa‐L1‐AD‐En2 significantly decreased *Cebpa* expression and adipocyte size, altered iWAT transcriptome, and affected iWAT development. We identified functional enhancers regulating *Cebpa* expression and clarified the crucial roles of Cebpa‐L1‐AD‐En2 and *Cebpa* promoter interaction in adipocyte differentiation and adipose tissue development.

## Graphical abstract

*Cebpa* is a master transcription factor gene for adipocyte differentiation and adipose tissue development. We employed circularized chromosome conformation capture sequencing (4C‐seq) technology to investigate chromatin interactions between *Cebpa* promoter and enhancers (PEIs) during adipogenesis. We identified functional enhancers regulating *Cebpa* expression in adipocyte differentiation and found that Cebpa‐L1‐AD‐En2 (En2) regulates adipocyte differentiation and adipose tissue development via the chromatin loop.

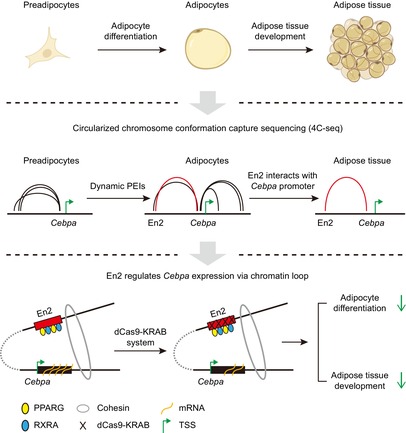

## 1 INTRODUCTION

Adipose tissue is a necessary energy storage and endocrine organ,^1^, ^2^ mainly comprising adipocytes. Adipocytes arise from mesenchymal stem cells via a cascade of events that involve adipocyte lineage commitment, proliferation, and differentiation.^3^, ^4^ The terminal differentiation of adipocytes is marked by distinct transcriptional changes, lipid droplet formation, and expression of adipogenic marker genes. This process is mainly regulated by CCAAT/enhancer binding protein alpha (CEBPA) and peroxisome proliferator‐activated receptor gamma (PPARG). CEBPA is a critical transcription factor (TF) in the second transcriptional wave of preadipocyte differentiation.^5^, ^6^, ^7^ CEBPA and PPARG cross‐regulate each other through positive feedback loops and transactivate downstream target genes, such as fatty acid‐binding protein 4 (*Fabp4*), adiponectin (*Adipoq*), lipoprotein lipase (*Lpl*), and solute carrier family 2 member 4 (*Slc2a4*, also known as *Glut4*).^8^, ^9^, ^10^, ^11^ CEBPA drives the terminal differentiation program of adipocytes and affects adipose tissue development. Studies show that CEBPA can efficiently promote the adipogenic program in various mouse fibroblastic cells and induce differentiation into mature adipocytes.^12^ *Cebpa* knockdown blocks the differentiation of 3T3‐L1 preadipocytes and prevents the accumulation of lipids.^13^, ^14^ Animal experiments show that *Cebpa* knockout mice die within 8 h of birth, and the adipocytes of these mice fail to accumulate lipids.^15^ Transgenic *Cebpa* ^−/−^ mice with liver‐specific expression of CEBPA at 7 days of age show an absence of white adipose tissue (WAT).^16^ Additionally, some studies have shown that genetic variation of *Cebpa* is associated with metabolic syndrome.^17^, ^18^, ^19^ These studies suggest that *Cebpa* plays an important role in adipocyte differentiation, adipose tissue development, and obesity‐related metabolic disease.

Mammalian genomes are non‐randomly folded in three‐dimensional (3D) nuclear space.^20^, ^21^ The 3D organization of the genome is complex and dynamic, and plays an essential role in regulating gene expression and cellular function.^22^, ^23^ It promotes or restricts chromatin interactions between enhancers and promoters to control gene regulation. Enhancers are important *cis*‐regulatory elements (CREs) that are scattered across the mammalian genome and frequently located in intergenic and intronic regions, but also in exonic regions.^24^, ^25^ Enhancers recruit cell type‐specific TFs in a sequence‐specific manner and have pivotal roles in the regulation of gene expression. Active enhancers are commonly marked by histone H3 lysine 27 acetylation (H3K27ac) and histone H3 lysine 4 monomethylation (H3K4me1) modifications, and typically feature p300 histone acetyltransferase binding and high chromatin accessibility.^26^, ^27^, ^28^, ^29^, ^30^ In vivo, distal enhancers located several kilobases (kb) or even megabases (Mb) away from their target genes are brought into proximity with their promoters through chromatin looping, which can be facilitated by architectural proteins, such as the cohesin complex (SMC1, SMC3, and RAD21), CCCTC binding factor (CTCF), and mediator complexes.^31^, ^32^, ^33^, ^34^ Interestingly, chromatin loops can regulate gene expression in a spatiotemporal and cell‐type‐specific manner, and represent a major mechanism of gene expression regulation. However, a detailed understanding of how chromatin loops regulate adipogenic gene expression is lacking.

Although many adipogenesis‐related enhancers have been identified by functional genomics,^35^, ^36^, ^37^, ^38^ the impact of enhancers on adipogenic gene expression and adipogenic differentiation is less well characterized. Since the discovery of the first enhancer to now,^39^ very little research has focused on enhancer‐mediated regulation of adipogenic gene expression. The first functional enhancer with specificity for adipose cells was identified in 1990, is located 5.4‐kb upstream of *Fabp4*, and is the primary determinant of expression of this gene in adipocytes in vivo.^40^ Direct binding to a distal enhancer is necessary for the transcriptional activation of human *Adipoq* by CEBPA.^41^ The 10‐kb enhancer upstream of the *Pparg2* locus forms a chromatin loop with the *Pparg2* promoter, binding the CEBPA and PRMT5 proteins to regulate *Pparg2* expression during adipogenic differentiation.^42^ Additionally, other studies have shown that silencing the BRD4‐occupied distal enhancer elements at the *Pparg* locus by CRISPR interference (CRISPRi) reduces *Pparg* gene expression and adipogenesis.^43^ These studies suggest that adipogenesis‐related enhancers play important roles in adipogenic gene expression and adipogenesis. CEBPA is a crucial TF that activates the expression of many adipocyte‐specific genes and is essential for adipocyte differentiation and adipose tissue development. Unravelling the transcriptional regulation of *Cebpa*, including its functional enhancers and the mechanisms by which they regulate *Cebpa* expression during adipocyte differentiation, will yield important insights into adipogenesis.

In this study, we employed circularized chromosome conformation capture sequencing (4C‐seq) to characterize dynamic 3D chromatin interactions of *Cebpa* during adipogenic differentiation. We identified the active enhancers of *Cebpa* in 3T3‐L1 adipocytes and functionally investigated their regulation of *Cebpa* expression and adipocyte differentiation. Transcriptome analysis showed that repression of one such enhancer, Cebpa‐L1‐AD‐En2, altered the adipocyte transcriptome and affected the adipogenic differentiation pathway. We demonstrated that the chromatin interaction between Cebpa‐L1‐AD‐En2 and the *Cebpa* promoter was regulated by cohesin. Furthermore, we found that RXRA and PPARG synergistically participate in Cebpa‐L1‐AD‐En2 activity. In vivo lentiviral injection into inguinal WAT (iWAT) showed that Cebpa‐L1‐AD‐En2 was important for *Cebpa* expression, adipocyte differentiation, and adipose tissue development. These findings will improve our understanding of enhancer function in adipogenesis. Additionally, understanding the molecular determinants of *Cebpa* expression and adipocyte differentiation may provide insights into the pathogenesis of metabolic diseases related to adipose tissue dysfunction.

## 2 MATERIALS AND METHODS

### 2.1 Ethics statement

All animal experiments were conducted according to the Regulations for the Administration of Affairs Concerning Experimental Animals (Ministry of Science and Technology, China, revised in March 2017) and approved by the Animal Ethical and Welfare Committee (AEWC) of Sichuan Agricultural University under permit No. DKY‐B2019102012.

### 2.2 Cell culture and adipocyte differentiation

Mouse embryo preadipocyte 3T3‐L1 cell line and human embryonic kidney (H293T) cell line were obtained from the Cell Bank of the Chinese Academy of Sciences (Shanghai, China). 3T3‐L1 and H293T cells were grown in Dulbecco's modified Eagle's medium (DMEM, Gibco) supplemented with 10% fetal bovine serum (FBS, Gibco) and 1% penicillin–streptomycin (Invitrogen) at 37°C in 5% CO_2_. Adipocyte differentiation of 3T3‐L1 cells was performed according to a standard adipogenic MDI cocktail (MDI: IBMX, dexamethasone, and insulin).^44^, ^45^ Briefly, 3T3‐L1 cells were seeded in 6‐well plates or T75 flasks. After reaching confluence, the cells were cultured for 48 h. Then, the cells were incubated in DMEM supplemented with an MDI cocktail (2 μg/mL of dexamethasone, 0.5 mM IBMX, and 10 μg/mL of insulin) and 10% FBS for 48 h. The medium with DMEM containing 10 μg/mL insulin and 10% FBS was changed every 2 days until the cells exhibited an adipocyte phenotype at Day 7 post‐induction. The differentiation efficiency of adipocytes was calculated as the ratio of cells stained with BODIPY 493/503 (differentiated cells) to the total number of cells stained with 4′,6‐diamidino‐2‐phenylindole (DAPI).

### 2.3 Oil red O staining

Oil red O powder (Sigma‐Aldrich) was dissolved in isopropanol. The stock solution was then diluted to 0.3% Oil red O solution with distilled H_2_O. Differentiated 3T3‐L1 cells were washed with phosphate‐buffered saline (PBS) and fixed in 4% formaldehyde at room temperature (RT) for 1 h. After fixation, cells were washed twice with purified water, then stained with Oil Red O solution for 1 h. Oil red O solution was removed, and the cells were washed with purified water. Stained cells were visualized by light microscopy, and the images were photographed using a microscope (Olympus, Tokyo, Japan). Six stained areas per well were randomly selected for the photograph.

### 2.4 RNA extraction, cDNA synthesis and reverse transcription and quantitative real‐time PCR

Total RNA was extracted with TRIzol reagent (Invitrogen) according to the manufacturer's protocols. The RNA (1 μg) was reverse‐transcribed into cDNA using the HiScript III RT SuperMix (Vazyme, China). The diluted cDNA was used as the template for quantitative real‐time PCR (qRT‐PCR). qRT‐PCR amplification was performed on the CFX Connect™ Real‐Time System (Bio‐Rad, USA) using the ChamQ Universal SYBR qPCR Master Mix (Vazyme, China). The reaction conditions were 95°C for 5 min, followed by 40 cycles at 95°C for 10 s and 60°C for 30 s. After PCR amplification, a melting curve was obtained: 95°C for 15 s, 60°C for 1 min, followed by 95°C for 15 s to verify primer specificity. Relative expression of genes was calculated using the 2^−ΔΔCt^ method^46^ after normalization to mRNA expression of the housekeeping gene *β*‐*actin*. All experiments were performed at least in triplicate. The qRT‐PCR primer sequences are summarized in Table S1.

### 2.5 4C‐seq library preparation and sequencing

4C‐seq was performed as described previously with some modifications.^47^, ^48^ For cultured cells, 1 × 10^7^ cells were crosslinked with fresh 2% formaldehyde for 10 min at RT. For adipose tissue, 1 g of frozen adipose tissue was crushed into powder using a mortar and pestle pre‐chilled in liquid nitrogen, and the powder was suspended in PBS and fixed with fresh 2% formaldehyde for 30 min at RT. Crosslinking was quenched with glycine to a final concentration of 0.13 M. Crosslinked cells were lysed for 15 min on ice in 10 mM Tris–HCl pH 8.0, 10 mM NaCl, and 0.2% NP‐40 supplemented with protease inhibitor. Nuclei were isolated by centrifugation and by removing the supernatant. The crosslinked chromatin was digested using primary restriction enzyme Dpn*II* (New England Biolabs) and religated by T4 DNA ligase (New England Biolabs) with rotation overnight at 16°C. After the reversal of crosslinking and RNA removal, DNA was extracted by phenol/chloroform and purified by ethanol precipitation. Purified DNA was digested using secondary restriction enzyme Nla*III* (New England Biolabs) and religated by T4 DNA ligase (New England Biolabs) overnight at 16°C. DNA was extracted by phenol/chloroform and purified using a QIAquick PCR cleanup kit. The DNA product served as the template for 4C PCR, and PCR was performed with a total amount of input of 3.2 μg and separated into 16 reactions. A two‐step PCR strategy was used to construct 4C‐seq sequencing library. PCR products were pooled and then run on 2% agarose gel. Smears from 200 to 800 bp were excised on the gel, and unwanted PCR product bands were removed. The 4C‐seq libraries were sequenced on an Illumina NovaSeq 6000 platform (Illumina) with 150‐bp paired‐end reads. The PCR primer sequences used in 4C‐seq are listed in Table S2.

### 2.6 Data analysis of 4C‐seq

Sequencing reads were demultiplexed, trimmed, and aligned using the pipe4C pipeline.^47^ 4C‐seq data analysis was performed using the pipe4C and r3Cseq package.^49^ In short, sequencing reads were demultiplexed based on the reading primer sequence of the viewpoint (first 20 nt of the sequence read). The reading primer sequences (not including the GATC Dpn*II* site) were trimmed from sequencing reads. The trimmed reads were mapped to the mouse (mm10) with bowtie2 (v2.2.5). The reads mapped to sequences containing the 4C primary restriction enzyme sites (Dpn*II*) and secondary restriction enzyme sites (Nla*III*) were termed 4C fragment‐ends (Frag‐ends). Non‐unique frag‐ends are discarded for posterior analysis. The generated SAM files were converted to BAM files, indexed, and sorted with Samtool (v1.11). Interaction regions of genome‐wide 2 kb‐window were identified using the r3Cseq by count and normalization of mapped reads, which was used to perform statistical analysis. Interaction sites with *q*‐value ≤ 0.05 were identified as significant interaction sites (SISs). 4C interaction profiles within ±200 kb of viewpoint were plotted by r3Cseq. The differential analysis of the interaction sites was performed using DESeq2 (v1.36.0) with the ‘ashr’ algorithm.^50^, ^51^ In addition, we also mapped 4C interaction profiles using pipe4C. The frag‐end with the highest coverage was removed from the dataset in the normalization process, and data were read‐depth normalized to 1 million aligned intra‐chromosomal reads. 4C‐seq coverage profile was obtained using “running means”, that is, coverage averages of 21 consecutive 4C frag‐ends.

### 2.7 Chromatin immunoprecipitation sequencing, DNAse I hypersensitivity sequencing, transposase‐accessible chromatin using sequencing, and global run‐on sequencing data processing and analysis

Publicly available chromatin immunoprecipitation sequencing (ChIP‐seq), DNAse I hypersensitivity sequencing (DHS‐seq), transposase‐accessible chromatin using sequencing (ATAC‐seq), and global run‐on sequencing (GRO‐seq) data were downloaded from the EBI ENA database (https://www.ebi.ac.uk/ena/browser/home). ChIP‐seq, DHS‐seq, and ATAC‐seq datasets were mapped to the mouse reference genome (mm10) with bowtie2 (v2.4.2) default parameters.^52^ PCR duplicates were removed with Samtool (v1.11) or Picard tools (v1.124) for further data analysis. Peak calling was carried out by MACS2 (v2.2.7.1) with a *q*‐value cut‐off of 0.05 as the threshold. Bigwig files were generated by the bedGraphToBigWig tool (v4) and used as input for the tracks in the figures. IGV (v2.10.0) was used for visualization purposes with bigwig files.

Analysis of the GRO‐seq data was carried out as previously described, with a few modifications.^53^, ^54^ Briefly, low‐quality bases, tailing polyA, and adapter sequences were trimmed using Cutadapt (v3.3). GRO‐seq reads were mapped to the mouse reference genome (mm10) using Bowtie (v1.0.0) with parameters ‘‐n 2 ‐l 32’. Uniquely mapped reads were used for downstream analysis. BedGraph files were generated using HOMER (v4.11) and converted into bigwig format using the bedGraphToBigWig tool (v4), and then bigwig files were visualized using IGV (v2.10.0). Detailed information on these data is provided in Table S3.

### 2.8 Identification of active enhancers of *Cebpa* gene

We set stringent criteria to identify active enhancers of *Cebpa*. First, we identified putative active enhancers of *Cebpa*. Genomic regions had obvious chromatin interaction with the *Cebpa* promoter and overlapped with H3K27ac, H3K4me1, and DHS‐seq peaks and at least ±1000 bp away from the TSS of known genes. These regions were defined as putative active enhancers of *Cebpa*. Second, we evaluated the activity of putative active enhancers using the dual luciferase reporter assay system. In brief, putative active enhancer regions (600–1300 bp) were PCR amplified using genomic DNA of 3T3‐L1 and cloned into SV40 promoter‐driven pGL3‐promoter luciferase reporter vector (Promega). On the third day after the induction of differentiation, differentiated 3T3‐L1 in 96‐well plates were transfected with pGL3–promoter–enhancer vector and Renilla luciferase vector using Lipofectamine 3000 (Invitrogen) according to the protocol provided by the manufacturer. The Renilla luciferase vector was co‐transfected as a control for normalizing luciferase activity. After 48 h of transfection, the reporter activity was measured by the Dual‐Glo luciferase assay kit (Promega, no. E2920) on a GloMax 96 microplate luminometer (Promega) according to the manufacturer's instructions. The genomic coordinates of putative active enhancers and the PCR primers of constructed pGL3–promoter–enhancer vectors are shown in Table S4.

### 2.9 Evolutionary conservation analysis of active enhancers

The sequence conservation of active enhancers was assessed using the phastCons method in the UCSC genome browser (http://genome-asia.ucsc.edu/). The conserved elements were analysed in 60 vertebrates. Each element is assigned a logarithm of the odds (LOD) scores equal to its log probability under the conserved model minus its log probability under the non‐conserved model. The ‘score’ contains transformed log‐odds scores, taking values between 0 and 1000. The raw log‐odds scores are retained in the ‘name.’ Elements conservation is measured as the LOD score of phastCons elements.

### 2.10 Single‐guide RNAs design and plasmid construction

The single‐guide RNAs (sgRNAs) were designed using CRISPOR (http://crispor.tefor.net/),^55^ CRISPR‐ERA (http://crispr-era.stanford.edu/),^56^ and CHOPCHOP (http://chopchop.cbu.uib.no/).^57^ All sgRNAs were examined for genome‐wide sequence specificity using CRISPR Finder (https://wge.stemcell.sanger.ac.uk/)^58^ and Cas‐OFFinder (http://www.rgenome.net/cas-offinder/).^59^ sgRNA target sites were selected for minimal predicted off‐target activity and maximal on‐target activity according to established algorithms. If the designed sgRNA sequence does not start with a ‘G’, a single ‘G’ nucleotide must be prepended to allow efficient transcription for the U6 promoter. Synthesized oligonucleotides were annealed and cloned into the BsmB*I*‐v2 restriction sites of pLV‐hU6‐sgRNA‐hUbC‐dCas9‐KRAB‐T2a‐Puro vector (Addgene, no. 71236) using DNA Ligation Kit (Takara, Japan). The sequences of all sgRNAs are listed in Table S5.

### 2.11 Lentivirus production, transduction and CRISPRi mediated repression of enhancers

H293T cells were cultured in T175 flask to produce lentivirus. When reached about 70% confluence, cells were co‐transfected simultaneously with 48 μg the pLV‐hU6‐sgRNA‐hUbC‐dCas9‐KRAB‐T2A‐Puro lentiviral expression plasmid (Addgene, no. 71236), 35 μg the second‐generation packaging plasmid psPAX2 (Addgene, no. 12260), and 12 μg envelope plasmid pVSV‐G (Addgene, 138479) using calcium phosphate cell transfection kit (Beyotime, C0508). After transfection 6–12 h, the medium was changed to fresh medium. Lentivirus‐containing supernatants were collected respectively at 24, 48, and 72 h post‐transfection and filtered by Millex‐HV 0.45 μm PVDF filters (Millipore, no. SLHV033RB). The viruses were further concentrated with 100 kDa Amicon Ultra‐15 centrifugal filter units (Amicon, no. UFC910008), and the titre was determined using a colloidal gold kit (Biodragon, Beijing, China). Concentrated viruses were stored at −80°C.

The 3T3‐L1 cells were plated in 12‐well plates overnight. When confluence reaches about 50%–60%, cells were infected with 1 mL lentiviruses medium containing dCas9‐KRAB‐En2‐sgRNAs (experimental group) or dCas9‐KRAB (control group) at the multiplicity of infection of 100 for 12 h. The medium was then replaced after 24 h post‐infection. At 48 h post‐infection, cells were selected with 2 μg/mL puromycin (Sigma). Untreated 3T3‐L1 and 3T3‐L1 stably expressing dCas9‐KRAB‐sgRNA or dCas9‐KRAB cells were induced to adipogenic differentiation using a standard MDI cocktail. dCas9‐KRAB system expressed in 3T3‐L1 cells was examined by qRT‐PCR.

### 2.12 Immunofluorescence staining and BODIPY 493/503 staining

Cells were fixed in 4% paraformaldehyde for 10 min. Cells were washed three times with PBS and permeabilized by treatment with 0.25% Triton X‐100 for 10 min. Cells were washed three times with PBS and blocked in 10% goat serum (Solarbio, SL038) for 40 min at RT. Cells were incubated with CEBPA antibody (Boster Biological Technology, A00386‐1) overnight at 4°C. Then, cells were washed three times with PBS and incubated with TRITC Conjugated AffiniPure Goat Anti‐rabbit IgG (H + L) secondary antibody (Boster Biological Technology, BA1090) at RT protected from light for 1 h. Finally, the nuclei were stained with DAPI staining solution (Beyotime, C1006) for 3 min at RT. To stain lipid droplets, 5 μM BODIPY 493/503 (Invitrogen, D3922) was applied to fixed cells for 20 min in the dark. Cells were washed three times with PBS and photographed with Zeiss Axio Observer 7 inverted fluorescent microscope.

### 2.13 RNA‐seq: Library preparation, sequencing, and data analysis

Total RNA was used as input material for the RNA sample preparations. Sequencing libraries were generated using NEBNext UltraTM RNA Library Prep Kit for Illumina (NEB). mRNA was purified from total RNA using poly‐T oligo‐attached magnetic beads. The library preparations were sequenced on the DNBSEQ‐T7 platform, and 150 bp paired‐end reads were generated. Sequence reads were aligned to the mouse reference genome (GRCm38/mm10) by HISAT2 (v2.2.1) and quantified by featureCounts from the Rsubread package (v2.8.1). Gene expression levels for each sample were calculated as transcripts per million values. Differentially expressed genes (DEGs) were identified using DESeq2 (v1.36.0). Functional enrichment analysis was performed using Metascape (a gene annotation & analysis resource, http://metascape.org/). GO/KEGG terms with *p*‐values ≤ 0.05 were considered significantly enriched. Gene set enrichment analysis (GSEA) was performed using GSEA version 4.2.3 software (https://www.gsea-msigdb.org/gsea/index.jsp) with MSigDBv7.5.1.

### 2.14 Small interfering RNA

Small interfering RNA (siRNA) duplexes were designed and synthesized by Sangon Biotechnology (Shanghai, China). siRNA oligos and negative control siRNA were transfected at a final concentration of 50 nM into 3T3‐L1 or 3T3‐L1 cells differentiated for 5 days using Lipofectamine 3000 (Invitrogen, L3000015) according to the manufacturer's protocol. Cells were harvested 48 h after transfection. The siRNA sequences are listed in Table S6.

### 2.15 Chromosome conformation capture assay

Quantitative analysis of chromosome conformation capture assays (3C‐qPCR) experiments were completed as described previously with minor modifications.^60^, ^61^ Briefly, 1 × 10^6^–1 × 10^7^ cells were crosslinked with 2% formaldehyde, while tumbling for 10 min at RT and quenched with a final concentration of 0.125 mM glycine for 15 ~ 20 min. Each aliquot of cells was lysed with 1 mL 1 × cold lysis buffer (50 mM Tris–HCl pH 8.0, 150 mM NaCl, 1% TritonX‐100, and 0.5% NP‐40 supplemented with protease inhibitor) for 10–15 min on ice. Nuclei were isolated by centrifugation and by removing the supernatant. Cell nuclei were pelleted, washed twice with 500 μL ice cold 1.2 × NEBuffer2.1 (New England Biolabs), and then re‐suspended in 500 μL ice cold 1.2 × NEBuffer2.1 with 0.3% sodium dodecyl sulfate (SDS) and incubated for 1 h at 37°C, followed by adding 2% Triton X‐100 and incubated for another 1 h to sequester the SDS. Each sample was digested overnight with 800 U of Hind*III* at 37°C. To stop the restriction digestion, 1.6% SDS (final concentration) was added, and samples were incubated at 65°C for 20–30 min. Ligation was performed overnight at 16°C in 50 mL tubes containing 6.125 mL 1.15 × T4 DNA Ligase Reaction Buffer (New England Biolabs), 1% Triton‐X 100 (final concentration), 1 mM ATP (final concentration), 1600 U T4 DNA ligase (final concentration). The crosslinks were reversed with Proteinase K (Sigma Aldrich) at 65°C overnight. 3C samples were then purified using phenol‐chloroform extraction. Purified DNA samples were used to perform qPCR with 250 ng DNA templates per 10 μL of qPCR reaction. Relative crosslinking frequency of the same site between different 3C samples was calculated using the value method^62^ and normalized to the values of the control *Ercc3* locus.^60^, ^63^, ^64^ Each qPCR reaction was performed in triplicate, and the data presented were the average of three independent experimental results for all PCR reactions. The Hind*III* digestion sites and 3C‐qPCR primer of the *Ercc3* locus are shown in Figure S4B. The primers of 3C‐qPCR are summarized in Table S7.

### 2.16 DNA pulldown assay and mass spectrometry

The enhancer region was amplified by PCR using primers having 5′‐desthio biotin modification, which was synthesized by Sangon Biotechnology (Shanghai, China). PCR products were gel‐purified using the QIAquick gel extraction kit (Qiagen). The concentration of the DNA was determined with Nanodrop 2000 spectrophotometer (Thermo Scientific). Differentiated 3T3‐L1 cells were cultured in T75 flasks and divided into the experimental and control groups, with each group having two replicates. The group containing biotin‐labelled DNA was the experimental group, and the group containing magnetic beads without DNA probe was the control group. The DNA pulldown assay was performed with a DNA pulldown kit (Catalogue Bes5004) according to the manufacturer's protocol (BersinBio, China). Briefly, the nuclear protein was extracted from differentiated 3T3‐L1 cells for Day 7. Streptavidin magnetic beads and desthio biotin‐labelled enhancer were incubated for 30 min at RT. Then, nuclear protein and DNA‐beads were incubated for 1 h with gentle agitation at 4°C to form DNA–protein‐beads complexes. The pulled‐down complex was washed four times with 800 μL ice‐cold binding buffer and eluted in 60 μL protein elution buffer for 2 h at 37°C. The pulled‐down proteins were analysed with a Q Exactive HF mass spectrometer (Thermo Fisher Scientific) coupled with an UltiMate 3000 RSLCnano UPLC system (Dionex). Mass spectrometry data were searched using MaxQuant (V2.1.2.0) and the Andromeda search engine against the UniProt mouse database (January 2022).

### 2.17 Transcription factor motif enrichment analysis

The enhancer sequence was downloaded from the NCBI database (https://www.ncbi.nlm.nih.gov/). Transcription factor‐binding site was predicted using the AnimalTFDB 3.0 (http://bioinfo.life.hust.edu.cn/AnimalTFDB#!/tfbs_predict)^65^ and JASPAR database (https://jaspar.genereg.net/).^66^ TF motifs with *p* < 0.05 were identified as enriched motifs. The sequence logo of TF‐binding sites motif was retrieved from the JASPAR database.

### 2.18 Construction of vectors and luciferase reporter assays

The coding sequence of *Pparg* and *Rxra* genes was cloned from cDNA templates of differentiated 3 T3‐L1 cells and inserted into pEGFP‐N1 by homologous recombination using a Trelief™ SoSoo Cloning Kit (Tsingke, TSV‐S2) according to the manufacturer's instructions. Motif‐mutant enhancers carrying the TF‐binding sites were amplified from 3T3‐L1 genomic DNA using homologous arm primers with motif mutations and cloned into the pGL3‐promoter vector by homologous recombination.

H293T cells were seeded in 96‐well plates. The cells were co‐transfected with a mixture of 50 ng PGL3‐promoter‐mutant enhancer, 10 ng Renilla, and 50 ng pEGFP‐N1‐PPARG/RXRA or pEGFP‐N1control using the Lipofectamine 3000. After 48 h of transfection, Firefly and Renilla luciferase activity was measured by the dual‐luciferase reporter assay system (Promega). Renilla luciferase activity served as an internal control, and firefly luciferase signals were normalized with Renilla luciferase signals. The primer sequences for vector construction are given in Table S4.

### 2.19 Injection of lentiviruses into inguinal adipose tissue in vivo

Six‐week‐old male and female C57BL/6J mice (*n* = 16) were housed in the animal facility were fed a high‐fat diet (HFD; TD.08811, 4.7 Kcal/g, ENVIGO) with water ad libitum for 1 week. C57BL/6J mice were divided into two groups (iWAT‐En2 group versus control group, *n* = 8 mice/group). dCas9‐KRAB‐En2 and dCas9‐KRAB‐GFP lentiviruses were generated using pLV‐hU6‐sgRNA‐hUbC‐dCas9‐KRAB‐T2a‐Puro (Addgene, no. 71236) and pLV‐hU6‐sgRNA‐hUbC‐dCas9‐KRAB‐T2a‐GFP (Addgene, no. 71237) plasmids, respectively, according to the above method for preparing lentivirus.

Seven‐week‐old female mice were anaesthetized with 1% pentobarbital sodium. According to previous methods,^67^, ^68^ 200 μL of lentivirus (5 × 10^7^ lentiviral transducing particles/mL) were injected directly into the iWAT of mice by multi‐point subcutaneous injection on each side. Dispersion of the injected volume into the iWAT using this procedure was validated using a trypan blue solution in the preliminary experiments (Figure S5A). Then, the mice were injected with lentivirus once every 3 days for four times injections. Each mouse was injected with a total of 800 μL lentivirus. iWAT‐En2 group mice were injected dCas9‐KRAB‐En2 lentiviruses, and the control group mice were injected dCas9‐KRAB‐GFP lentiviruses. After injection, mice were fed ad libitum HFD until sacrifice. Green fluorescent protein (GFP) expression of iWAT from control group mice was detected with anti‐GFP primary antibody (Abcam, ab290) and goat anti‐rabbit DyLight 488 secondary antibody (Boster Biological Technology, BA1127). Pictures were taken using Zeiss Axio Observer 7 inverted fluorescent microscope. At 6 weeks post‐injection, mice were euthanized for further experiments. The iWAT and body weight of mice were weighed. The expression level of *Cebpa* gene in iWAT was detected by qRT‐PCR. The adipocyte diameter and area of the iWAT from control group and iWAT‐En2 group mice were measured after haematoxylin and eosin (H&E) staining of paraffin sections (5‐μm thick) using Image‐Pro Plus 6.0.

### 2.20 Statistical analysis

Statistical analyses were conducted in SPSS Statistics 19.0. All data shown were determined for three independent experiments unless otherwise stated and presented as the mean ± standard deviation (SD). Differences were considered statistically significant at *p < 0.05.

## 3 RESULTS

### 3.1 Characterization of *Cebpa* chromatin interactomes during adipocyte differentiation

To investigate chromatin interactions of *Cebpa* during adipocyte differentiation, we performed 4C‐seq analyses with the *Cebpa* promoter (−2200 bp to +500 bp of the transcription start site) as the viewpoint in 3T3‐L1 preadipocytes (3T3‐L1‐PRE) and 3T3‐L1 adipocytes (3T3‐L1‐AD; Figure S1A). Differentiation of 3T3‐L1‐PRE was induced using a standard adipogenic cocktail.^44^, ^45^ The 3T3‐L1 preadipocyte cell line is widely used as an adipocyte differentiation model system for studying the molecular mechanisms of adipogenesis.^69^, ^70^, ^71^ We observed numerous lipid droplets of different sizes in 3T3‐L1 cells differentiated for 7 days compared with no droplets in 3T3‐L1‐PRE (Figure S1B). The expression levels of *Cebpa* and adipogenic marker genes (*Pparg*, *Srebf1*, *Adipoq*, *Fabp4*, and *Slc2a4*) were significantly up‐regulated (*p* < 0.05) after differentiation (Figure S1C). These results suggested that we had successfully induced adipogenic differentiation of 3T3‐L1 cells and obtained 3T3‐L1 adipocytes.

**FIGURE 1:**
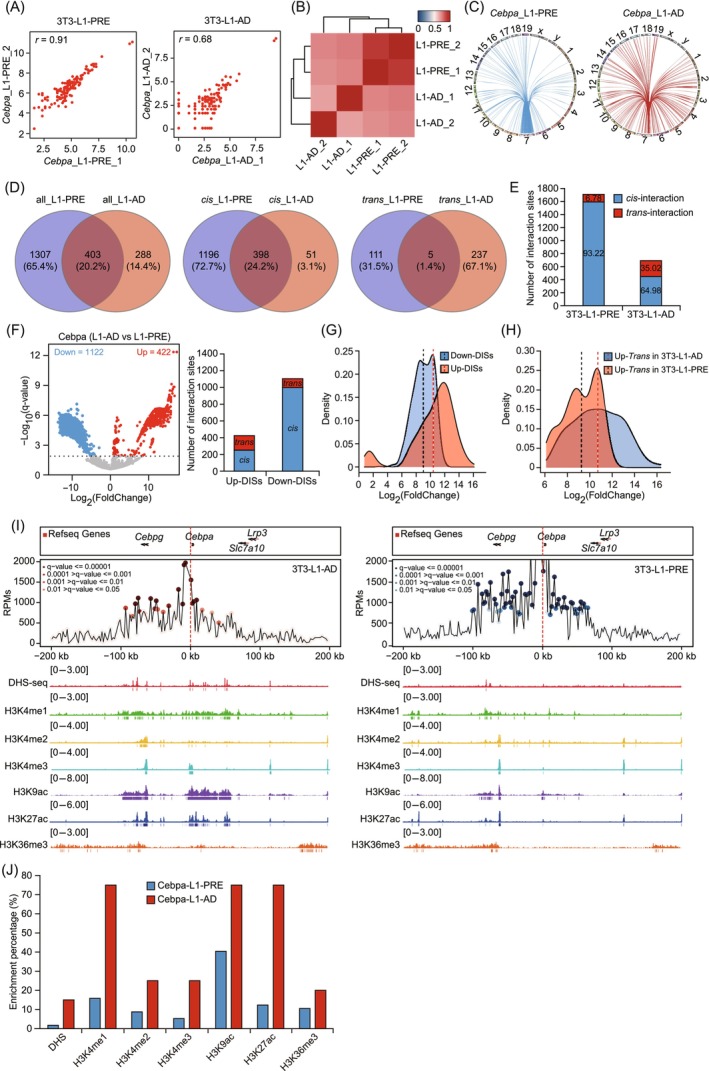
Identification of genome‐wide chromatin interactions of *Cebpa* in 3T3‐L1 preadipocytes (3T3‐L1‐PRE) and 0033T3‐L1 adipocytes (3T3‐L1‐AD). (A) Scatter plot showing chromatin interactions of *Cebpa*. The number of interaction sites (in Log_2_) in each genomic bin (1 Mb *cis*) in two replicates was plotted. The Pearson's correlation coefficient is shown in the panel. (B) Heatmap showing the clustering of chromatin interactions of *Cebpa*. The colour scale indicates the correlation degree: blue, low correlation; red, high correlation. (C) Circos plot depicting *cis*‐ and *trans*‐interactions of *Cebpa*. Chromosomes are shown in a circular orientation, with numbers and letters above the circle indicating the name of each. (D) Venn diagram showing the number of overlapping and unique chromatin sites of *Cebpa*. (E) Numbers of reliable interaction sites and ratios of *cis*−/*trans*‐interaction sites of *Cebpa*. Numbers inside or above the column indicate the percentage of *cis*‐/*trans*‐interaction sites. (F) Volcano plot showing differential interaction sites (DISs) of *Cebpa* between 3T3‐L1‐PRE and 3T3‐L1‐AD (left). The threshold of DISs in the volcano plot was ‐Log_10_(*q*‐value) ≥ 2; red and blue dots represent up‐regulated and down‐regulated DISs in 3T3‐L1‐AD, respectively. Number of *cis*‐/*trans*‐DISs between 3T3‐L1‐PRE and 3T3‐L1‐AD (right). (G) Density plot showing the |Log_2_(FoldChange)| distribution of up‐/down‐regulated DISs for *Cebpa*; vertical red‐ and black‐dashed lines indicate the mean values of |Log_2_(FoldChange)| for up‐regulated and down‐regulated DISs, respectively. (H) Density plot showing the |Log_2_(FoldChange)| distribution for up‐regulated *trans*‐DISs in 3T3‐L1‐AD and 3T3‐L1‐PRE; vertical red‐ and black‐dashed lines indicate the mean values of |Log_2_(FoldChange)| in 3T3‐L1‐AD and 3T3‐L1‐PRE, respectively. (I) Changes in chromatin state at significant interaction sites (SISs) of *Cebpa* before and after differentiation. Upper panel: Chromatin interactions of the *Cebpa* promoter generated by r3Cseq analysis. Red circles represent SISs in 3T3‐L1‐AD; blue circles represent SISs in 3T3‐L1‐PRE. The dotted red line represents the viewpoint of *Cebpa*. Lower panel: DNAse I hypersensitivity sequencing (DHS‐seq) and chromatin immunoprecipitation sequencing (ChIP‐seq) profiles of the *Cebpa* locus in 3T3‐L1‐PRE and 3T3‐L1‐AD. (J) Enrichment analysis of epigenetic modifications at the SISs. Bar plots show the percentage of SISs enriched with epigenetic modifications in 3T3‐L1‐PRE and 3T3‐L1‐AD.

Next, we performed 4C‐seq experiments and analysed the 4C data (two replicates each 3T3‐L1‐PRE and 3T3‐L1‐AD, respectively) using the pipe4C pipeline^47^ and r3Cseq,^49^ respectively. We obtained between 10.68 and 24.59 million filtered, aligned reads per 4C dataset (median, 17.9 million reads), with 76.42%–81.89% of the total reads in the four 4C datasets distributed on the *cis*‐chromosome (Figure S1D). Our 4C data conformed to the ‘*cis*/overall ratio of >40%’ criteria proposed by van de Werken et al.,^48^ indicating that experiments were of good quality. Additionally, the four datasets showed that >40% of fragment ends within 100 kb of the viewpoint had at least one read (Figure S1D), indicating that our 4C libraries were of high complexity and contained enough information to draw reproducible conclusions.^47^ Detailed quality metrics are given in Table S8. We identified the genome‐wide chromatin interactions of *Cebpa* in a continuous non‐overlapping 2‐kb window for each 4C dataset (Table S9) and evaluated the reproducibility of chromatin interactions between replicates by counting the number of *cis*‐interactions in every 1‐megabase (Mb) genomic region. The Pearson's correlation coefficients of *Cebpa* in 3 T3‐L1‐PRE and 3 T3‐L1‐AD were 0.91 and 0.68, respectively (Figure 1A), indicating good consistency between replicates. Clustering analysis showed that replicates of 3T3‐L1‐PRE and 3T3‐L1‐AD were not well separated into two clusters (Figure 1B); however, this result also suggested some degree of discrepancy in the chromatin interaction patterns of *Cebpa* between 3T3‐L1‐PRE and 3T3‐L1‐AD.

To study changes in chromatin interactions of *Cebpa* during differentiation, we first identified reliable interaction sites between replicates in a continuous non‐overlapping 2‐kb window. This revealed 1710 and 691 reliable interaction sites of *Cebpa* in 3T3‐L1‐PRE and 3T3‐L1‐AD, respectively, after overlapping replicates (Figure 1C and Table S10). There were 403 shared interaction sites in 3T3‐L1‐PRE and 3T3‐L1‐AD, including 398 *cis*‐interaction sites and 5 *trans*‐interaction sites (Figure 1D). Additionally, we found that *Cebpa* in 3T3‐L1‐PRE had more than twice the number of interaction sites as *Cebpa* in 3T3‐L1‐AD, with up to 90% of the interaction sites located on the *cis*‐chromosome; in 3T3‐L1‐AD, only 64.98% of the interaction sites were located on the *cis*‐chromosome (Figure 1E). More *trans*‐interaction sites were observed in 3T3‐L1‐AD (242, 35.02%) than in 3T3‐L1‐PRE (116, 6.78%), suggesting that *Cebpa* increases *trans*‐interactions after differentiation. A differential analysis of interaction sites between 3T3‐L1‐PRE and 3T3‐L1‐AD using DESeq2^50^, ^51^ was used to identify differential interaction sites (DISs) with a *q*‐value ≤ 0.01. We found 1544 DISs between 3T3‐L1‐AD and 3T3‐L1‐PRE, accounting for 77.28% (1544/1998) of all interaction sites (Figure 1F and Table S11). In 3T3‐L1‐AD, there were 422 up‐regulated DISs and 1122 down‐regulated DISs, and 1256 *cis*‐DISs and 288 *trans*‐DISs (Figure 1F). Density plots were used to examine the fold change distributions for up‐regulated/down‐regulated DISs. The |Log_2_(FoldChange)| values of up‐regulated and down‐regulated DISs showed different distributions (Figure 1G). Most up‐regulated DISs had larger fold changes than most down‐regulated DISs, with mean |Log_2_(FoldChange)| values of 10.55 and 9.13, respectively. This suggested that the up‐regulated DISs of *Cebpa* in 3T3‐L1‐AD had a stronger change in interaction frequency than the up‐regulated DISs of *Cebpa* in 3T3‐L1‐PRE. Then, we also examined the fold change distribution for *trans*‐ and *cis‐*DISs of up‐regulated DISs in 3T3‐L1‐AD and 3T3‐L1‐PRE. The |Log_2_(FoldChange)| values of up‐regulated *trans*‐DISs in 3T3‐L1‐AD and 3T3‐L1‐PRE showed different distributions (Figure 1H). Similarly, the |Log_2_(FoldChange)| values of up‐regulated *cis‐*DISs also showed different distributions in 3T3‐L1‐AD and 3T3‐L1‐PRE (Figure S1E). The mean |Log_2_(FoldChange)| values of *trans*‐ and *cis‐*DISs in 3 T3‐L1‐AD were larger than those in 3T3‐L1‐PRE (Figures 1H and S1E). These results highlight changes and differences in *Cebpa* chromatin interactions during adipogenic differentiation.

Significant interaction sites (SISs) identified by r3Cseq represent potential structural or functional interaction sites with important regulatory roles in gene expression. We identified a total of 77 SISs, 57 in 3T3‐L1‐PRE and 20 in 3T3‐L1‐AD (Table S10). The SISs were all located within ±200 kb of the *Cebpa* promoter (Figure 1I), implying that these regions might have potential for regulation of *Cebpa* expression. Analysis revealed 19 shared SISs in 3T3‐L1‐PRE and 3T3‐L1‐AD, 38 unique SISs in 3T3‐L1‐PRE, and one unique SIS in 3T3‐L1‐AD. Changes in chromatin interaction during adipogenic differentiation are often accompanied by changes in chromatin state.^72^, ^73^ To look for changes in the SIS chromatin state of *Cebpa* before and after differentiation, we analysed epigenetic modifications, including six histone modifications (H3K4me1, H3K4me2, H3K4me3, H3K9ac, H3K27ac, and H3K36me3) and DNase I hypersensitivity sites (DHSs) at the SISs. We found that, in 3T3‐L1‐AD, a higher proportion of SISs was enriched in all seven of these epigenetic marks than in 3T3‐L1‐PRE (Figure 1J and Table S12), consistent with previous studies showing that promoters of highly expressed genes interact with regions that are highly enriched in active histone marks. This finding also suggested that the chromatin state of SISs in 3T3‐L1‐AD is more open after differentiation, and that these open chromatin regions include CREs. Overall, our results indicated that dynamic epigenetic modification of *Cebpa* interaction sites occurred during differentiation.

### 3.2 Identification and evolutionary conservation analysis of *Cebpa* active enhancers

**FIGURE 2:**
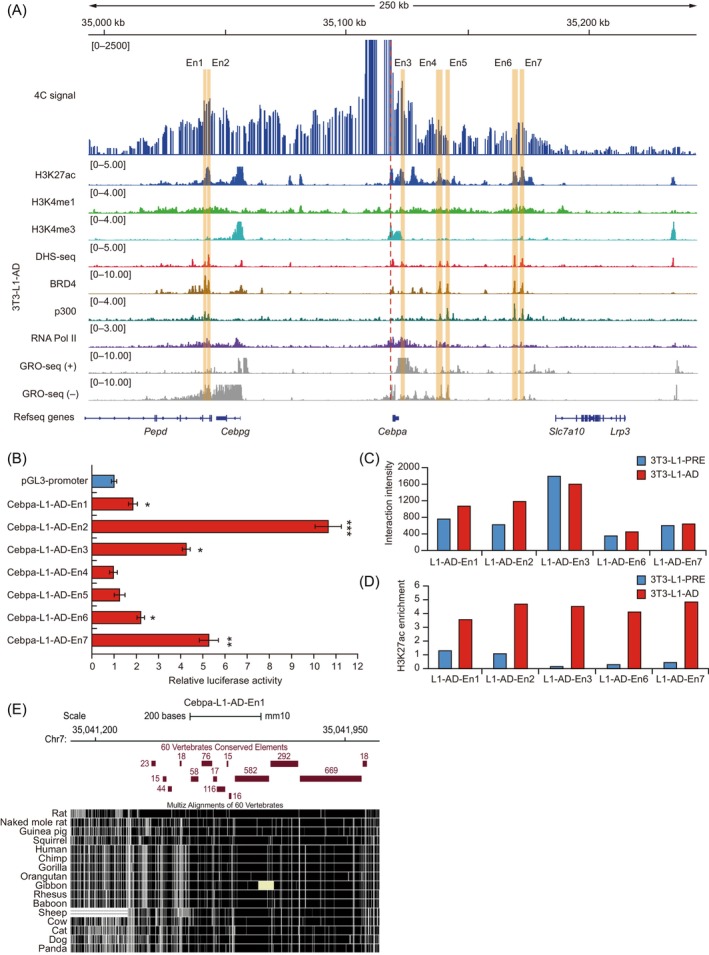
Identification of *Cebpa* active enhancers in 3T3‐L1 adipocytes (3T3‐L1‐AD). (A) Integrative Genomics Viewer (IGV) screenshot showing the manual selection of putative active enhancers of *Cebpa* in 3T3‐L1‐AD. The upper track shows circularized chromosome conformation capture coupled with next‐generation sequencing (4C‐seq) interaction profiles of *Cebpa* in 3T3‐L1 differentiated for 7 days; 4C signals of replicates are merged. Lower tracks show ChIP‐seq profiles of marks H3K27ac, H3K4me1, H3K4me3, BRD4, p300, and RNA Pol II, and DNAse I hypersensitivity sequencing (DHS‐seq) and global run‐on sequencing (GRO‐seq) profiles at *Cebpa* loci in 3T3‐L1‐AD. The red‐dotted line represents the viewpoint of *Cebpa*. The orange column represents the putative active enhancers. (B) Luciferase reporter assays of *Cebpa* enhancer activity. pGL3–promoter–enhancer vector was transfected to 3T3‐L1 cells differentiated for 3 days, and luciferase activity was detected 2 days after transfection. The pGL3–promoter was used as a control. Firefly luciferase signals were normalized with Renilla luciferase signals. Data expressed as mean ± SD of three independent experiments, and *p*‐values were calculated using Student's *t*‐test; *p < 0.05, **p < 0.01, ***p < 0.001. Chromatin interaction intensity (C) and H3K27ac histone enrichment (D) of active enhancer regions in 3T3‐L1‐PRE and 3T3‐L1‐AD using data obtained from the IGV. (E) Sequence conservation analysis of Cebpa‐L1‐AD‐En1 in selected species using the UCSC genome browser. Horizontal red bars indicate conserved elements in 60 vertebrates. Element conservation was measured as logarithm of the odds scores of phastCons program elements.

Enhancers are important CREs in the genome that specifically activate gene transcription to regulate gene expression.^25^, ^39^, ^74^ To identify active enhancers of *Cebpa*, we analysed publicly available active enhancer‐associated ChIP‐seq of H3K27ac and H3K4me1 and DHS‐seq datasets of 3T3‐L1‐PRE and 3T3‐L1‐AD. Using the criteria for active enhancers described in Section 2, we identified three putative active enhancers in 3T3‐L1‐PRE and seven putative active enhancers in 3T3‐L1‐AD (Figures 2A and S2A). Two of the putative active enhancers were shared by 3T3‐L1‐PRE and 3T3‐L1‐AD: Cebpa‐L1‐En2 corresponds to Cebpa‐L1‐AD‐En1, and Cebpa‐L1‐En3 corresponds to Cebpa‐L1‐AD‐En2. Eight putative active enhancer regions were significantly enriched in both H3K27ac and H3K4me1 (marks of active enhancers), but not in H3K4me3 (mark of active promoters), with chromatin accessibility (DHS‐seq), coactivator p300, and RNA polymerase II (RNA Pol II) peaks (Figures 2A and S2A). BRD4 is an epigenetic reader enriched in active enhancers and promoters, and is essential for adipogenesis.^38^ Thus, we also analysed BRD4 ChIP‐seq data and observed that these putative enhancer regions were markedly enriched with BRD4 peak signals (Figures 2A and S2A). These results indicated that the candidate regions displayed obvious characteristics of active enhancers.

To assess the enhancer activity of *Cebpa*, we performed luciferase reporter assays for the seven candidate enhancers of 3T3‐L1‐AD in differentiated 3T3‐L1 cells. We found that five of the seven enhancers (Cebpa‐L1‐AD‐En1, ‐En2, ‐En3, ‐En6, and ‐En7) significantly increased (*p* < 0.05) luciferase activity compared with the pGL3‐promoter vector (Figure 2B). Notably, Cebpa‐L1‐AD‐En2 showed the strongest transcriptional activity of the seven enhancers, with >10‐fold higher luciferase activity than the pGL3‐promoter vector. Cebpa‐L1‐AD‐En3 and ‐En7 exhibited higher transcriptional activities than Cebpa‐L1‐AD‐En1 and ‐En6, and they increased 4.25‐ and 5.70‐fold luciferase activity than the pGL3‐promoter vector, respectively. This result indicated that five of the *Cebpa* enhancers were active, corresponding to two intronic regions (Cebpa‐L1‐AD‐En1 and ‐En2) and three intergenic regions (Cebpa‐L1‐AD‐En3, ‐En6, and ‐En7). To assess the potential differential activity of the five enhancers during adipogenic differentiation, we repeated the luciferase reporter assays of each enhancer in 3T3‐L1 preadipocytes. Cebpa‐L1‐AD‐En1 and ‐En2 showed significantly increased (*p* < 0.05) luciferase activity compared with the pGL3‐promoter vector in 3T3‐L1 preadipocytes. In contrast, Cebpa‐L1‐AD‐En3, ‐En6, and ‐En7 did not increased luciferase activity in 3T3‐L1 preadipocytes (Figure S2B). These results indicated that the activity of *Cebpa* enhancers changes in a stage‐specific manner during adipogenic differentiation. GRO‐seq, a widely used method for measuring nascent RNA,^75^, ^76^ showed bidirectional transcription at these active enhancer regions, especially in the Cebpa‐L1‐AD‐En2 region (Figure 2A). We then analysed differences in chromatin interactions and histone modifications of the five enhancer regions between 3 T3‐L1‐PRE and 3 T3‐L1‐AD. Except for the region of Cebpa‐L1‐AD‐En3, the interaction intensity at the other enhancer regions increased after differentiation, particularly at Cebpa‐L1‐AD‐En2 (Figures 2C and S2C). Additionally, H3K27ac and H3K4me1 histone modifications in these enhancer regions significantly increased after differentiation, especially H3K27ac (Figures 2D and S2). This result suggested that chromatin interaction and histone modification might be important for the functioning of these active enhancers, possibly affecting *Cebpa* expression during adipocyte differentiation.

Enhancers usually have evolutionary conservation properties.^77^, ^78^, ^79^, ^80^, ^81^, ^82^, ^83^ We used the UCSC genome browser (http://genome-asia.ucsc.edu/, GRCm38/mm10) to perform conservation analysis of the 5 active enhancers in 60 vertebrates. All of the active enhancers contained at least four conserved elements (Figures 2E and S2D), measured as LOD scores of phastCons program elements. Cebpa‐L1‐AD‐En1 showed the highest sequence conservation of the five active enhancers (Figure 2E). The sum of LOD scores of Cebpa‐L1‐AD‐En1, ‐En2, and ‐En3 was >350, and at least one conserved element in each of these enhancers had an LOD score ≥200 (Figures 2E and S2D). These results suggested that the five active enhancers of *Cebpa* were evolutionarily conserved.

### 3.3 Identification of functional enhancers regulating *Cebpa* expression and adipocyte differentiation

**FIGURE 3:**
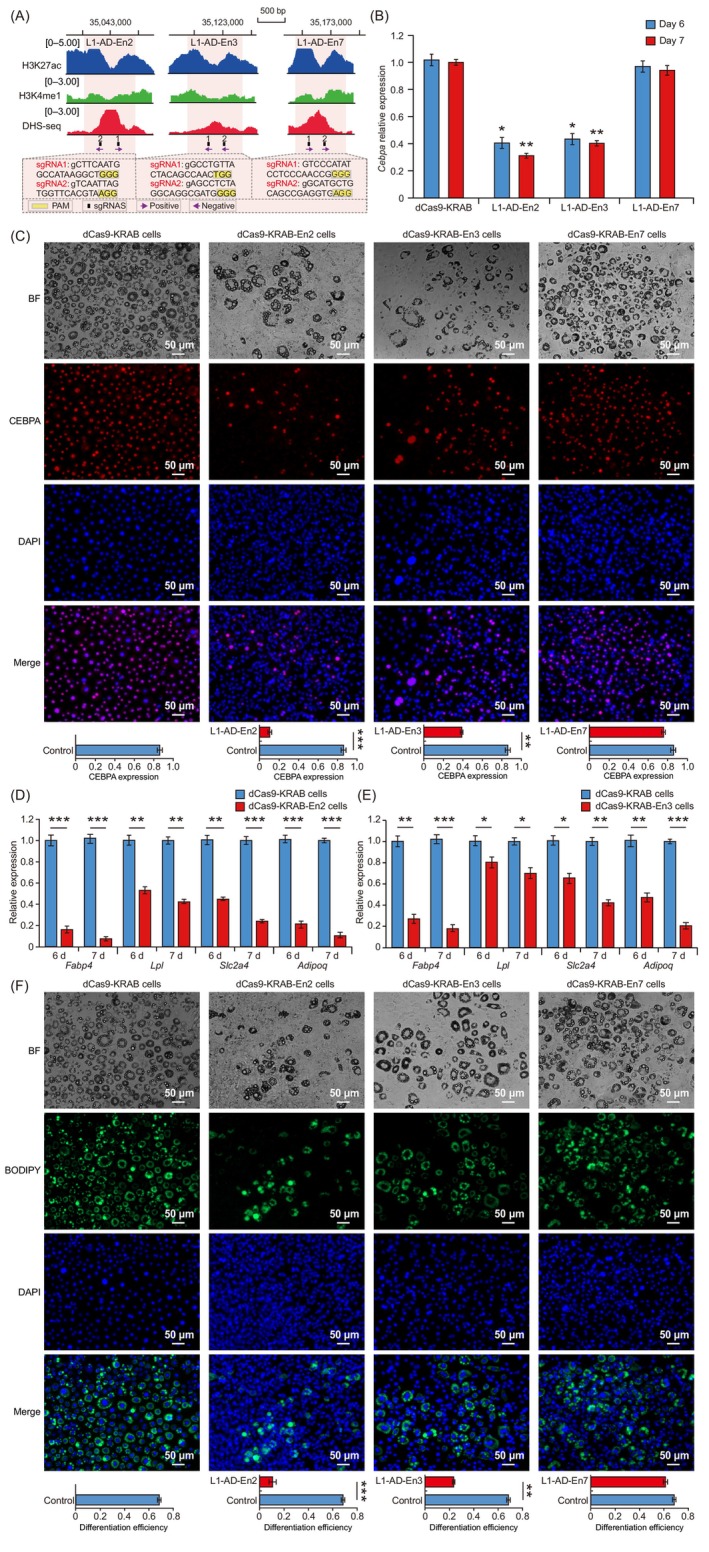
Regulation of *Cebpa* expression and adipocyte differentiation by Cebpa‐L1‐AD‐En2 and ‐En3. (A) The dCas9‐KRAB system modifies the chromatin of the Cebpa‐L1‐AD‐En2, ‐En3, and ‐En7 regions. ChIP‐seq of active enhancer‐associated histone modification (H3K27ac, H3K4me1) and DNAse I hypersensitivity sequencing (DHS‐seq) profiles show the chromatin status of the enhancer regions. Enhancer regions are shown as orange rectangles. Single‐guide RNA (sgRNA) target locations are indicated in black with corresponding black numbers; arrow direction indicates targeting of the forward or reverse DNA strand. (B) Quantitative real‐time PCR (qRT‐PCR) analysis of *Cebpa* expression in 3T3‐L1 transduced with dCas9‐KRAB, dCas9‐KRAB‐En2‐sgRNAs, dCas9‐KRAB‐En3‐sgRNAs, or dCas9‐KRAB‐En7‐sgRNAs and induced to differentiate for Days 6 and 7. Data expressed as mean ± standard deviation (SD; *n* = 3). (C) Immunofluorescence staining of CEBPA in 3T3‐L1 transduced with dCas9‐KRAB, dCas9‐KRAB‐En2‐sgRNAs, dCas9‐KRAB‐En3‐sgRNAs, or dCas9‐KRAB‐En7‐sgRNAs and induced to differentiate for 7 days; BF (bright field), CEBPA (red), and 4′,6‐diamidino‐2‐phenylindole (DAPI, blue). Quantification of CEBPA‐positive nuclei in dCas9‐KRAB cells and dCas9‐KRAB‐sgRNA cells by counting five randomly selected microscopic fields. qRT‐PCR analysis of CEBPA downstream target genes (*Fabp4*, *Lpl*, *Slc2a4*, and *Adipoq*) in 3T3‐L1 transduced with dCas9‐KRAB‐En2‐sgRNAs (D) or dCas9‐KRAB‐En3‐sgRNAs (E) at days 6 and 7 of differentiation. Data expressed as mean ± SD (*n* = 3). (F) BODIPY (green) staining of lipid droplets in 3T3‐L1 transduced with dCas9‐KRAB, dCas9‐KRAB‐En2‐sgRNAs, dCas9‐KRAB‐En3‐sgRNAs, or dCas9‐KRAB‐En7‐sgRNAs at Day 7 of differentiation; DAPI (blue). The differentiation efficiency of dCas9‐KRAB and dCas9‐KRAB‐sgRNA cells was determined by counting cells in five randomly selected microscopic fields. The *p*‐values were calculated using Student's *t*‐test; *p < 0.05, **p < 0.01, ***p < 0.001.

To investigate whether the identified active enhancers regulate *Cebpa* expression during adipocyte differentiation, we used the dCas9‐KRAB epigenetic system (a CRISPR repressor system) to study the top three enhancers with the highest fluorescence activities. We designed two sgRNAs per enhancer region (Figure 3A and Table S5), packaged lentivirus, infected 3T3‐L1‐PRE, and generated 3T3‐L1‐PRE stably expressing the dCas9‐KRAB control or dCas9‐KRAB‐sgRNA system (Figure S3A,B). qRT‐PCR experiments were performed to assess the expression of *Cebpa* mRNA at Days 6 and 7 of differentiation. We found that *Cebpa* expression was significantly repressed (*p* < 0.05) on Days 6 and 7 by targeting Cebpa‐L1‐AD‐En2 or ‐En3 regions (Figure 3B). CRISPRi targeting of the Cebpa‐L1‐AD‐En2 and ‐En3 regions significantly repressed *Cebpa* expression by 69% and 60%, respectively, compared with dCas9‐KRAB control at Day 7 of differentiation (Figure 3B). Repression of the Cebpa‐L1‐AD‐En7 region did not significantly decrease *Cebpa* expression at Day 7 of differentiation (Figure 3B). Immunofluorescence staining of CEBPA was performed in 3T3‐L1 expressing dCas9‐KRAB, dCas9‐KRAB‐En2‐sgRNAs, dCas9‐KRAB‐En3‐sgRNAs or dCas9‐KRAB‐En7‐sgRNAs. Targeted repression of Cebpa‐L1‐AD‐En2 and ‐En3 reduced the fluorescence intensity of CEBPA protein compared with that under dCas9‐KRAB control at Day 7 of differentiation (Figure 3C), indicating that the repression of these enhancers impaired CEBPA expression. In contrast, Cebpa‐L1‐AD‐En7 repression did not significantly change the fluorescence intensity of CEBPA protein compared with that under dCas9‐KRAB control at Day 7 of differentiation (Figure 3C). The immunofluorescence staining results were consistent with the mRNA expression levels of *Cebpa*. Next, we examined the expression of CEBPA downstream target genes. qRT‐PCR analysis showed that Cebpa‐L1‐AD‐En2 repression led to significant down‐regulation (*p* < 0.01) of *Fabp4*, *Lpl*, *Slc2a4*, and *Adipoq* compared with that under dCas9‐KRAB control at Days 6 and 7 of differentiation (Figure 3D). Repression of Cebpa‐L1‐AD‐En3 also led to significant down‐regulation (*p* < 0.05) of *Fabp4*, *Lpl*, *Slc2a4*, and *Adipoq* at Days 6 and 7 of differentiation (Figure 3E). Additionally, BODIPY staining results indicated that repression of Cebpa‐L1‐AD‐En2 and ‐En3 attenuated lipid droplet formation and blocked adipocyte differentiation (Figure 3F). These results indicated that Cebpa‐L1‐AD‐En2 and ‐En3, as functional enhancers, regulate *Cebpa* expression and adipocyte differentiation.

**FIGURE 4:**
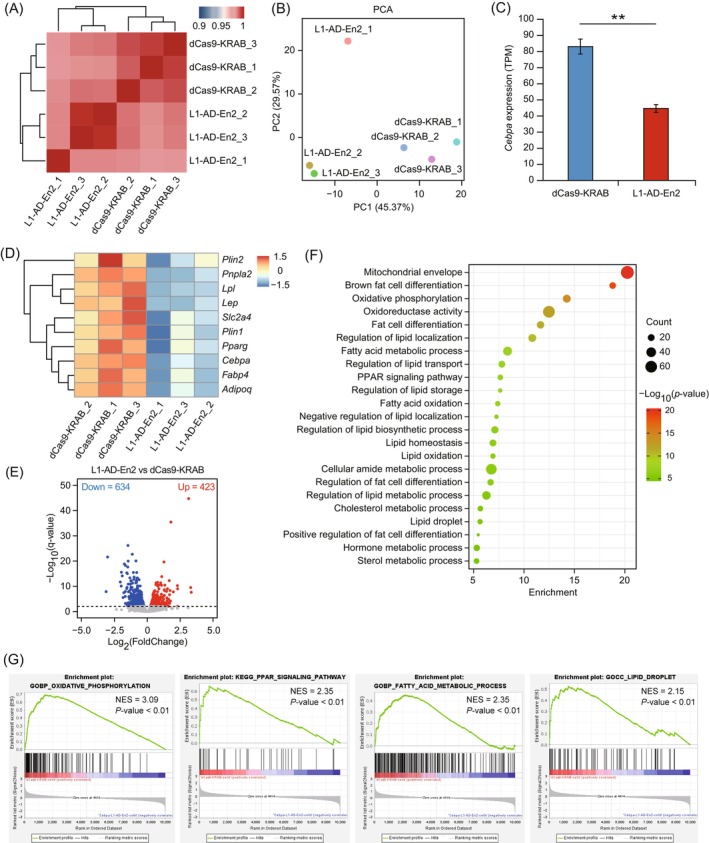
Effects of Cebpa‐L1‐AD‐En2 repression on the transcriptome during adipogenic differentiation. (A) Heatmap showing hierarchical clustering of the Pearson's correlation scores (represented by colour) between samples of dCas9‐KRAB‐En2 and dCas9‐KRAB cells. (B) Principal component analysis (PCA) plot of normalized RNA‐seq data of dCas9‐KRAB‐En2 and dCas9‐KRAB cells expressed as transcripts per million (TPM). Percentages shown on each axis represent the percentages of variation explained by the principal components. (C) Expression levels (TPM) of *Cebpa* in dCas9‐KRAB and dCas9‐KRAB‐En2 cells. Bars show the mean and standard deviation (*n* = 3); **p < 0.01. (D) Heatmap showing the expression levels of genes (z‐scores) in dCas9‐KRAB and dCas9‐KRAB‐En2 cells. Genes were subjected to hierarchical clustering. (E) Volcano plot showing differentially expressed genes (DEGs, *q*‐value ≤ 0.01) between dCas9‐KRAB and dCas9‐KRAB‐En2 cells. Blue and red dots indicate DEGs up‐regulated in dCas9‐KRAB‐En2 and dCas9‐KRAB cells, respectively. (F) Functional enrichment analyses of down‐regulated DEGs in dCas9‐KRAB‐En2 cells by Metascape. The dot size represents the number of genes, and the colour bar represents the ‐Log_10_(*p*‐value). (G) Gene set enrichment analysis of all expressed genes in dCas9‐KRAB and dCas9‐KRAB‐En2 cells. A positive value of the normalized enrichment score (NES) indicates enrichment in dCas9‐KRAB cells, and a negative value indicates enrichment in dCas9‐KRAB‐En2 cells.

To explore the effect of enhancer repression on adipocyte differentiation in a transcriptome‐wide manner, we performed transcriptional profiling by RNA sequencing (RNA‐seq) of dCas9‐KRAB‐En2 or dCas9‐KRAB cells at Day 7 of differentiation. Hierarchical clustering and principal component analysis (PCA) showed that there was high correlation between replicate samples and differences in transcriptome profiles between dCas9‐KRAB‐En2 and dCas9‐KRAB cells (Figure 4A,B). RNA‐seq analysis showed that *Cebpa* expression was significantly lower (*p* < 0.01) in dCas9‐KRAB‐En2 cells than in dCas9‐KRAB cells (Figure 4C), which was consistent with the qRT‐PCR analysis of *Cebpa* transcription (Figure 3B). Expression levels of *Pparg*, known CEBPA downstream target genes (*Fabp4*, *Slc2a4*, *Adipoq*, *Lep*, and *Lpl*), lipid droplet‐associated genes (*Plin1* and *Plin2*), and the adipocyte triglyceride lipase gene (*Pnpla2*) were significantly downregulated (*p* < 0.01) in dCas9‐KRAB‐En2 compared with those in dCas9‐KRAB cells (Figure 4D). Differential expression analysis of dCas9‐KRAB‐En2 and dCas9‐KRAB cells identified a total of 1057 DEGs (*q*‐value ≤ 0.01), including 634 down‐regulated and 423 up‐regulated genes (Figure 4E and Table S13). Functional enrichment of DEGs was carried out using the Metascape website (http://metascape.org/gp/index.html#/main/step1). Functional enrichment analysis of down‐regulated DEGs in dCas9‐KRAB‐En2 cells revealed significant enrichment of terms related to oxidation, adipocyte differentiation, lipid droplet metabolism and lipid transport, and storage (Figure 4F), including the mitochondrial envelope, oxidative phosphorylation, fat cell differentiation, lipid oxidation, and regulation of lipid biosynthetic process, lipid transport and lipid storage. Additionally, GSEA of expressed genes showed significant enrichment of oxidative phosphorylation, peroxisome proliferator‐activated receptor (PPAR) signalling pathway, fatty acid catabolic process, lipid droplet, triglyceride biosynthetic process, and fat cell differentiation in dCas9‐KRAB cells (Figures 4G and S3C). These results showed that Cebpa‐L1‐AD‐En2 repression alters the transcriptome of adipocytes and inhibits adipocyte differentiation and lipid synthesis pathways, demonstrating that Cebpa‐L1‐AD‐En2 is critical for regulating *Cebpa* transcription and adipogenesis.

Cebpa‐L1‐AD‐En2 is located in intron 13 of *Pepd*, which is expressed in 3T3‐L1‐PRE and 3T3‐L1‐AD (Figure S3D,E). Targeting Cebpa‐L1‐AD‐En2 with the dCas9‐KRAB system may interfere with the expression of *Pepd*. A previous study showed that *Pepd* plays a vital role in collagen turnover by degrading proline‐containing dipeptides, and dysregulation of macrophage PEPD in obesity determines adipose tissue fibro‐inflammation and insulin resistance.^84^ Whether *Pepd* perturbation affects the expression of *Cebpa* during adipocyte differentiation remains unclear. To investigate this question, we used siRNA to silence *Pepd* expression in 3T3‐L1‐PRE and 3T3‐L1‐AD. The results showed that a ~70% decrease in the expression of *Pepd* (*p* < 0.05) did not significantly affect the expression of *Cebpa* (*p* > 0.05) in 3T3‐L1‐PRE or 3T3‐L1‐AD compared with that in the control group (Figure S3F). Moreover, the expression levels of CEBPA downstream target genes, including *Fabp4*, *Lpl*, *Adipoq*, and *Slc2a4*, did not significantly change (*p* > 0.05) in 3 T3‐L1 differentiated for 7 days compared with the control group (Figure S3G). These results suggested that *Pepd* does not regulate *Cebpa* expression during adipocyte differentiation.

### 3.4 A cohesin‐mediated chromatin loop is responsible for Cebpa‐L1‐AD‐En2 and *Cebpa* promoter chromatin interaction

**FIGURE 5:**
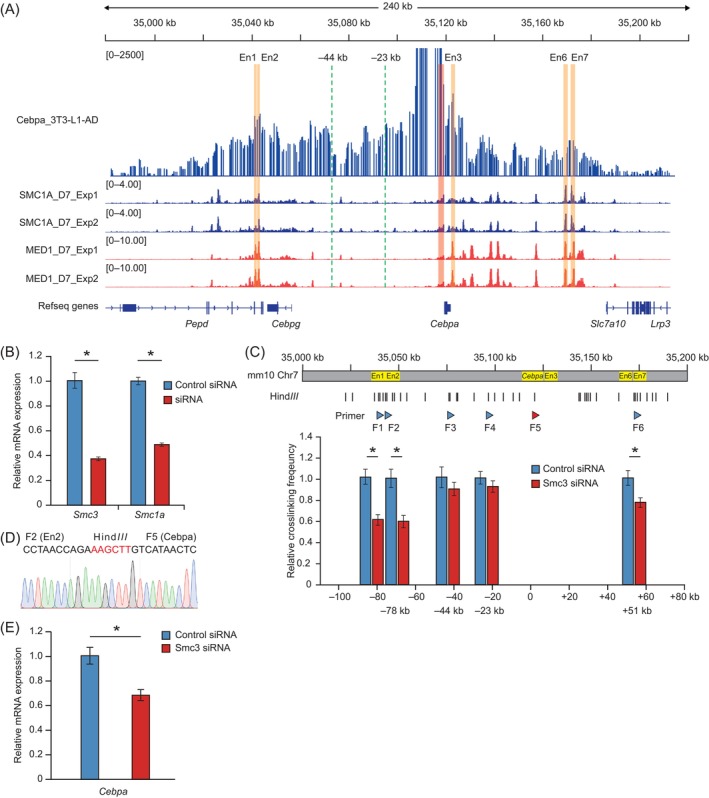
Cohesin‐mediated regulation of the chromatin interaction of Cebpa‐L1‐AD‐En2 and the *Cebpa* promoter. (A) Upper track shows the circularized chromosome conformation capture coupled with next‐generation sequencing (4C‐seq) interaction profile of *Cebpa* in 3T3‐L1 differentiated for 7 days. Lower tracks show ChIP‐seq profiles of SMC1A (blue) and MED1 (red) at *Cebpa* loci in differentiated 3T3‐L1 cells. The orange column represents the Cebpa‐L1‐AD‐En2 region, the red column represents the *Cebpa* promoter region, and the green dotted line represents the genomic sites without cohesin enrichment. (B) Quantitative real‐time PCR (qRT‐PCR) analysis of *Smc3* and *Smc1a* in differentiated 3T3‐L1 cells transfected with control, *Smc3*, or *Smc1a* small interfering RNA (siRNA). (C) Chromatin interactions between the *Cebpa* promoter and other chromatin sites (−80, −78, −44, and +51 kb) in differentiated 3T3‐L1 assessed by quantitative analysis of chromosome conformation capture (3C‐qPCR) 2 days after transfection with control or *Smc3* siRNA. Chromatin interactions at the same genomic locus in different cells were normalized to the *Ercc3* locus. The triangle represents the 3C‐qPCR primer, and the arrow direction represents the primer direction. Yellow rectangles indicate sites of cohesin enrichment. (D) Sanger sequencing of 3C‐qPCR product between the *Cebpa* promoter and Cebpa‐L1‐AD‐En2 (−78 kb site). The red base indicates the Hind*III* digestion site formed. (E) Relative expression of *Cebpa* measured by qRT‐PCR in differentiated 3T3‐L1 cells transfected with control or *Smc3* siRNA. All data expressed as mean ± standard deviation (*n* = 3). The *p*‐values were calculated using Student's *t*‐test; *p < 0.05.

Cohesin‐mediated chromatin loops play a pivotal role in gene expression by regulating enhancer–promoter interactions.^32^, ^85^, ^86^ We investigated whether the chromatin interaction between Cebpa‐L1‐AD‐En2 and the *Cebpa* promoter is regulated by cohesin‐mediated chromatin loops. Studies have shown that genes are preferentially regulated by enhancers within the same topologically associating domain (TAD).^87^, ^88^, ^89^, ^90^, ^91^ CTCF, as an insulator‐binding protein, is often found to be often enriched in TAD boundaries.^92^ Thus, we first analysed published high‐throughput chromosome conformation capture data and CTCF ChIP‐seq data in 3T3‐L1‐AD. We found that Cebpa‐L1‐AD‐En2 and *Cebpa* are located in an interaction domain in which the boundaries are enriched for CTCF binding and that the CTCF binding motif shows a convergent orientation (arrows indicate direction and strand; Figure S4A). This result suggested that Cebpa‐L1‐AD‐En2 and the *Cebpa* promoter are located within a TAD. Next, we analysed ChIP‐seq data for the cohesin subunit SMC1A, which is essential for forming a cohesin‐mediated chromatin loop, and found significant enrichment of SMC1A peaks in the Cebpa‐L1‐AD‐En2 and *Cebpa* promoter regions (Figure 5A). Additionally, Med1, a subunit of the complex that mediates chromatin loops between enhancers and promoters,^34^, ^93^ showed significant binding at the Cebpa‐L1‐AD‐En2 and *Cebpa* promoter regions (Figure 5A). These results implied that a cohesin‐mediated chromatin loop regulates the chromatin interaction between Cebpa‐L1‐AD‐En2 and *Cebpa* promoter.

To examine the potential role of cohesin in the chromatin interaction of Cebpa‐L1‐AD‐En2 and the *Cebpa* promoter, we depleted the cohesin subunits SMC3 and SMC1A in 3T3‐L1‐AD using siRNA. qRT‐PCR analysis showed that, after siRNA transfection, the expression levels of *Smc3* and *Smc1a* had decreased (*p* < 0.05) by 63% and 51% at Day 7 of differentiation, respectively (Figure 5B). Quantitative analysis of chromosome conformation capture (3C‐qPCR) assays was performed in differentiated 3 T3‐L1 transfected with *Smc3* siRNA or control siRNA. A schematic of the *Cebpa* locus depicting active enhancers, cohesin binding sites, and Hind*III* digestion sites, together with the corresponding primers used for 3C analyses, is provided in Figure 5C. The 3C‐qPCR analysis indicated that *Smc3* knockdown markedly reduced (*p* < 0.05) the chromatin interactions of the *Cebpa* region with the −78 kb (Cebpa‐L1‐AD‐En2) sites compared with those in control cells (Figure 5C). Whereas the interactions at the −44 and −23 kb sites without cohesin enrichment did not change markedly compared with those in control cells. In addition, we also found that the chromatin interactions at the −80 kb (Cebpa‐L1‐AD‐En1) and +51 kb site (Cebpa‐L1‐AD‐En7) containing cohesin enrichment also decreased significantly (Figure 5C). The 3C ligation product of the −78 kb site was validated by Sanger sequencing (Figure 5D). These results suggested that cohesin depletion changed the chromatin interaction between Cebpa‐L1‐AD‐En2 and the *Cebpa* promoter region. Additionally, we evaluated the expression of *Cebpa*. Notably, *Cebpa* expression was significantly down‐regulated (*p* < 0.05) in *Smc3*‐depleted 3T3‐L1 differentiated for 7 days (Figure 5E). Taken together, our results indicated that cohesin is required for the chromatin loop between Cebpa‐L1‐AD‐En2 and the *Cebpa* promoter, and contributes to regulating *Cebpa* expression.

### 3.5 RXRA and PPARG synergistically regulate the activity of Cebpa‐L1‐AD‐En2

**FIGURE 6:**
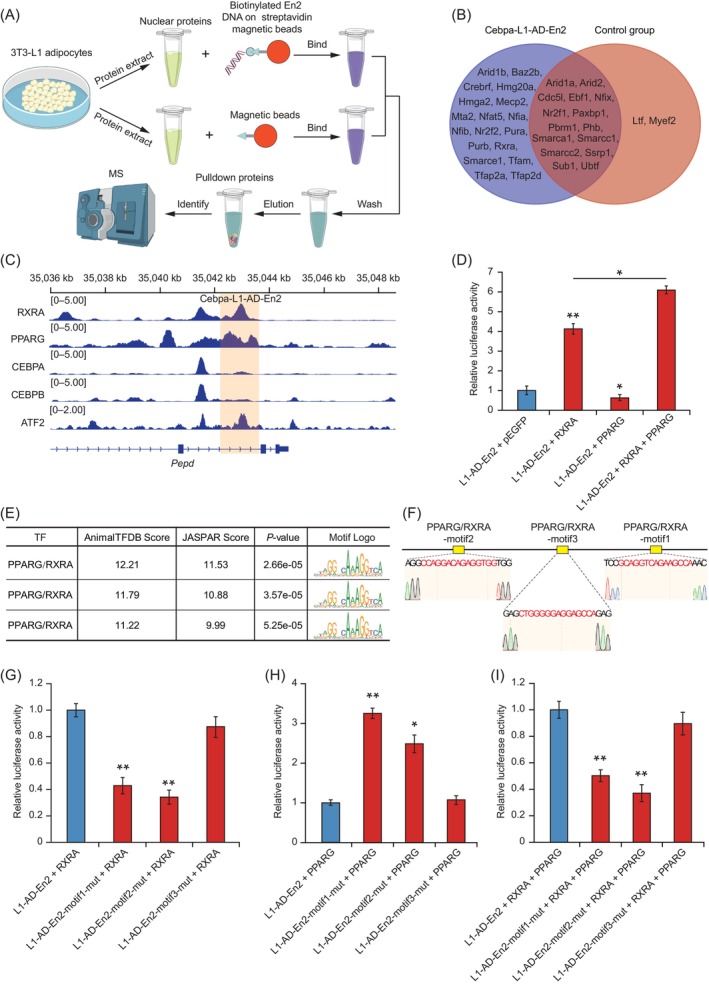
Regulation of Cebpa‐L1‐AD‐En2 activity by transcription factors (TFs) RXRA and PPARG. (A) Schematic overview of DNA pulldown assay used to determine TF binding to the Cebpa‐L1‐AD‐En2 region. (B) Venn diagram showing the shared and unique TFs of the Cebpa‐L1‐AD‐En2 and control group identified using the DNA pulldown assay in two replicates of 3T3‐L1 differentiated for 7 days. The Cebpa‐L1‐AD‐En2 group, containing biotin‐labelled En2 DNA and magnetic bead, was set as the experimental group. The control group contained magnetic beads without the DNA probe. (C) ChIP‐seq profiles showing binding of RXRA, PPARG, CEBPA, CEBPB, and ATF2 to the Cebpa‐L1‐AD‐En2 region. (D) Relative luciferase activity of Cebpa‐L1‐AD‐En2 detected under co‐transfection with plasmids overexpressing RXRA (50 ng), PPARG (50 ng), or RXRA (25 ng)/PPARG (25 ng) in H293T cells. Data expressed as mean ± standard deviation (SD) of three independent experiments. (E) PPARG/RXRA heterodimer motif enrichment analysis in the Cebpa‐L1‐AD‐En2 region. (F) Schematic diagram showing the location of predicted PPRAG/RXRA heterodimer binding sites and motif sequence in the Cebpa‐L1‐AD‐En2 region, and Sanger sequencing confirmation of the deletion mutations of each motif. (G–I) Relative luciferase activity of Cebpa‐L1‐AD‐En2 regions carrying PPARG/RXRA heterodimer binding site mutations (motif1, motif2, and motif3) detected under RXRA (50 ng), PPARG (50 ng), or RXRA (25 ng)/PPARG (25 ng) overexpression in H293T cells. Data expressed as mean ± SD of three independent experiments. The *p*‐values were calculated using Student's *t*‐test; *p < 0.05, **p < 0.01.

Transcription factors bind to enhancers through specific DNA sequences and regulate their activity, affecting target gene expression under the enhancer and promoter interaction.^94^, ^95^, ^96^, ^97^ We investigated which TFs control the activity of Cebpa‐L1‐AD‐En2 beginning with an in vitro DNA pulldown assay using nuclear lysates of 3T3‐L1‐AD coupled with mass spectrometric analysis (Figure 6A). We identified 18 TFs that showed specificity for binding to the Cebpa‐L1‐AD‐En2 region in vitro compared with the control group (Figure 6B and Table S14). Notably, we found that retinoid X receptor (RXR)‐like family proteins (RXRA and NR2F2) and CAAT box TF/nuclear factor I (CTF/NFI) family proteins (NFIA and NFIB) bound to the Cebpa‐L1‐AD‐En2 region (Figure 6B). Previous studies have shown these TFs to be involved in adipogenesis and energy metabolism,^98^, ^99^ suggesting that they may provide the specific activity of Cebpa‐L1‐AD‐En2. We also detected binding of the high‐mobility group A family member HMGA2, which modifies chromatin structure by interacting with the transcriptional machinery to regulate gene transcription.^100^, ^101^, ^102^ Next, we searched the available ChIP‐seq data of adipogenesis‐related TFs (RXRA, PPARG, CEBPA, CEBPB, and activating TF 2 [ATF2]) from the CistromeDB (http://cistrome.org/db/#/) and analysed the binding of these TFs to the Cebpa‐L1‐AD‐En2 region in 3T3‐L1‐AD. ChIP‐seq analysis revealed significant binding of RXRA to the Cebpa‐L1‐AD‐En2 region in the endogenous chromatin context (Figure 6C), which agreed with our in vitro pulldown results (Figure 6B). We observed minimal binding of CEBPA and CEBPB, and obvious binding of ATF2, to the Cebpa‐L1‐AD‐En2 region (Figure 6C). ATF2 contributes to the regulation of adipocyte differentiation and function.^103^ Additionally, we noted that PPARG, a critical TF in adipocyte differentiation, bound strongly to the Cebpa‐L1‐AD‐En2 region in 3 T3‐L1‐AD (Figure 6C). Previous studies have shown that PPARG binds to PPAR response elements (PPREs) as an obligate heterodimer with RXRA to activate adipocyte gene expression and regulate adipocyte differentiation.^104^, ^105^, ^106^ Based on this, we speculated that RXRA and PPARG may regulate the activity of Cebpa‐L1‐AD‐En2.

To verify this hypothesis, we performed luciferase reporter assays in H293T cells transfected with pGL3‐promoter‐En2 and RXRA and PPARG overexpression plasmids. Compared with transfection of the pGL3‐promoter‐En2 plasmid only, Cebpa‐L1‐AD‐En2 activity increased 4‐fold under co‐transfection with the RXRA overexpression plasmid (Figure 6D). In contrast, Cebpa‐L1‐AD‐En2 activity was repressed upon co‐transfection with the PPARG overexpression plasmid (Figure 6D). A 1.5‐fold increase in Cebpa‐L1‐AD‐En2 activity was observed upon co‐transfection of both the RXRA and PPARG expression plasmids compared with the RXRA expression plasmids, suggesting a synergistic effect between RXRA and PPARG on the Cebpa‐L1‐AD‐En2 activity (Figure 6D). Previous studies have shown that, in the absence of ligands, PPARG binds to PPREs and recruits corepressor complexes that mediate transcription repression.^107^, ^108^, ^109^, ^110^ However, in the presence of active RXRA, PPARG does not interact with these corepressors, and instead forms PPARG/RXRA permissive heterodimers to activate transcription of target genes.^111^, ^112^, ^113^ Therefore, this result suggests that RXRA and PPARG synergistically regulate the activity of Cebpa‐L1‐AD‐En2, possibly as a heterodimer.

To further investigate whether the key PPARG/RXRA heterodimer binding site affect the activity of the Cebpa‐L1‐AD‐En2, we performed TF binding site motif analysis using the JASPAR^66^ and AnimalTFDB3.0^65^ databases. Three co‐predicted PPARG/RXRA heterodimer binding sites were identified and named PPARG/RXRA‐motif1, ‐motif2, and ‐motif3 (Figure 6E,F). We then constructed pGL3‐promoter‐En2 plasmids with each deletion mutations of each binding site (Figure 6F) and performed luciferase reporter assays under co‐transfection with RXRA, PPARG, or PPARG/RXRA overexpression plasmids in H293T cells. The PPARG/RXRA‐motif2 and ‐motif1 mutations significantly reduced the activity of Cebpa‐L1‐AD‐En2 upon co‐transfection of either motif mutant plasmid with the RXRA overexpression plasmid, whereas the PPARG/RXRA‐motif3 mutation did not significantly change Cebpa‐L1‐AD‐En2 activity (Figure 6G). Under co‐transfection with the PPARG overexpression plasmid, the PPARG/RXRA‐motif1 and ‐motif2 mutations significantly increased Cebpa‐L1‐AD‐En2 activity, while the PPARG/RXRA‐motif3 mutation had no significant effect on Cebpa‐L1‐AD‐En2 activity (Figure 6H). This result indicated that PPARG/RXRA‐motif1 and ‐motif2 are essential for the activity of RXRA‐ and PPARG‐regulated Cebpa‐L1‐AD‐En2. Next, we cotransfected the pGL3‐promoter‐En2‐motif‐mutant plasmid with the RXRA/PPARG overexpression plasmid. Consistent with the results of RXRA overexpression, the PPARG/RXRA‐motif1 and ‐motif2 mutations significantly reduced Cebpa‐L1‐AD‐En2 activity, while the PPARG/RXRA‐motif3 mutation did not (Figure 6I). These results suggested that RXRA and PPARG directly regulate the activity of Cebpa‐L1‐AD‐En2 by binding to the motif1 and motif2 sites of PPARG/RXRA heterodimer.

### 3.6 Repression of Cebpa‐L1‐AD‐En2 decreases Cebpa expression in iWAT and affects adipose tissue development

**FIGURE 7:**
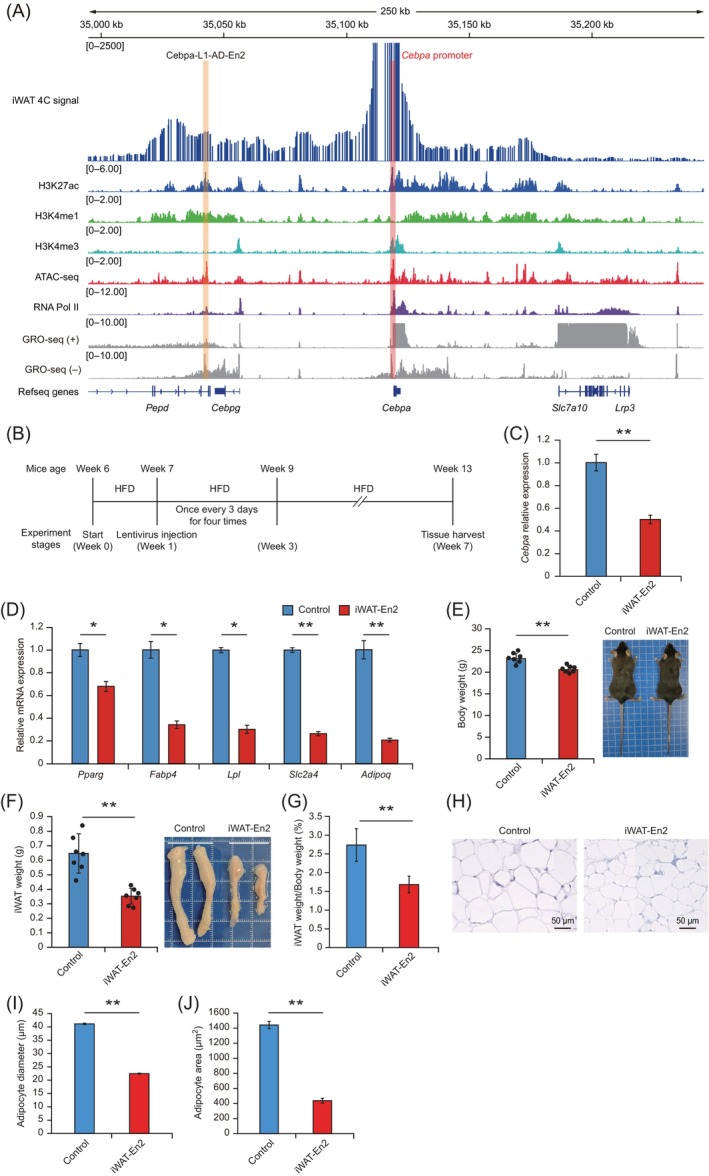
Regulation of adipocyte size and adipose tissue development by Cebpa‐L1‐AD‐En2. (A) Active enhancer Cebpa‐L1‐AD‐En2 interaction with the *Cebpa* promoter in mouse inguinal adipose tissue (iWAT). The upper track shows the circularized chromosome conformation capture coupled with next‐generation sequencing (4C‐seq) chromatin interaction profile of *Cebpa* in iWAT. Lower tracks show the chromatin immunoprecipitation sequencing profiles of H3K27ac, H3K4me1, H3K4me3, and RNA Pol II peaks, and the assay for transposase‐accessible chromatin using sequencing (ATAC‐seq) and global run‐on sequencing (GRO‐seq) profiles at *Cebpa* loci in iWAT. (B) Schematic of experimental design of lentiviral injection into iWAT. iWAT‐En2 group mice were injected dCas9‐KRAB‐En2 lentiviruses, and the control group mice were injected dCas9‐KRAB‐GFP lentiviruses. Control and iWAT‐En2 group mice were fed a high‐fat diet (HFD). (C) Quantitative real‐time PCR analysis of *Cebpa* expression in iWAT of En2 and control mice. Data expressed as mean ± standard deviation (SD; *n* = 7). (D) Relative expression of *Pparg*, *Fabp4*, *Lpl*, *Slc2a4*, and *Adipoq* in iWAT of iWAT‐En2 and control mice. Data expressed as mean ± SD (*n* = 7). Body weight (E), iWAT weight (F), and ratio of iWAT weight to body weight (G) of iWAT‐En2 and control mice. Data expressed as mean ± SD (*n* = 7 for each group). (H) Representative haematoxylin and eosin staining image of iWAT of iWAT‐En2 and control mice; scale bars, 50 μm. Quantitative analysis of adipocyte diameter (I) and area (J) in En2 and control mice (*n* = 7 groups of 700 adipocytes used to calculate adipocyte diameter and area). Data expressed as mean ± standard error of the mean (*n* = 7). The *p*‐values were calculated using Student's *t*‐test; *p < 0.05, **p < 0.01.

To investigate whether Cebpa‐L1‐AD‐En2 produces a marked effect on adipogenesis in mouse adipose tissue, we injected the dCas9‐KRAB‐En2 lentiviral system directly into iWAT.^67^, ^68^ First, we performed a 4C‐seq experiment on iWAT and analysed publically available ChIP‐seq for H3K27ac, H3K4me1 and RNA Pol II, as well as ATAC‐seq and GRO‐seq datasets in mouse WAT to evaluate whether the chromatin interaction between Cebpa‐L1‐AD‐En2 and the *Cebpa* promoter identified in 3T3‐L1‐AD also exists in mouse adipose tissue. We found that the *Cebpa* promoter had strong chromatin interactions with the Cebpa‐L1‐AD‐En2 region (Figure 7A). The Cebpa‐L1‐AD‐En2 region was enriched with active enhancer marks (H3K27ac and H3K4me1) and exhibited high chromatin accessibility (ATAC‐seq peaks; Figure 7A). We also observed that the ChIP‐seq signal of RNA Pol II was enriched in the Cebpa‐L1‐AD‐En2 region (Figure 7A). Additionally, GRO‐seq analysis showed significant enrichment of RNA Pol II signals in the Cebpa‐L1‐AD‐En2 region, especially on the negative DNA strand (Figure 7A), suggesting that the Cebpa‐L1‐AD‐En2 region has the potential to transcribe enhancer RNAs (eRNAs). These results suggested that the Cebpa‐L1‐AD‐En2 region is important for regulation of *Cebpa* expression in mouse adipose tissue.

Next, we performed lentiviral injection of the iWAT outlined in Figure 7B. At 2 weeks post‐injection, immunofluorescence microscopy analysis revealed expression of GFP in inguinal cells of control mice (Figure S5B). *Cebpa* expression levels in iWAT from iWAT‐En2 group mice and control group mice were analysed by qRT‐PCR. Mice with repression of Cebpa‐L1‐AD‐En2 showed markedly decreased (*p* < 0.01) *Cebpa* expression in iWAT compared with that in control group mice at 13 weeks of age (Figure 7C). The expression levels of *Pparg*, *Fabp4*, *Lpl*, *Slc2a4*, and *Adipoq* in iWAT were significantly lower (*p* < 0.05) in iWAT‐En2 group mice than in control group mice at 13 weeks of age (Figure 7D). Following up on these results, we performed phenotypic analyses of iWAT between iWAT‐En2 group and control group mice. Compared with the control group mice, iWAT‐En2 group mice show significant decreases (*p* < 0.01) in body weight (Figure 7E), iWAT weight (Figure 7F), and the ratio of iWAT weight to body weight (Figure 7G). H&E staining indicated that the adipocytes in iWAT from dCas9‐KRAB‐En2 mice were smaller (diameter and area) than those from control mice (Figures 7H–J and S5C). These results suggested that the repression of Cebpa‐L1‐AD‐En2 reduces *Cebpa* expression and adipocyte size, and affects iWAT development.

**FIGURE 8:**
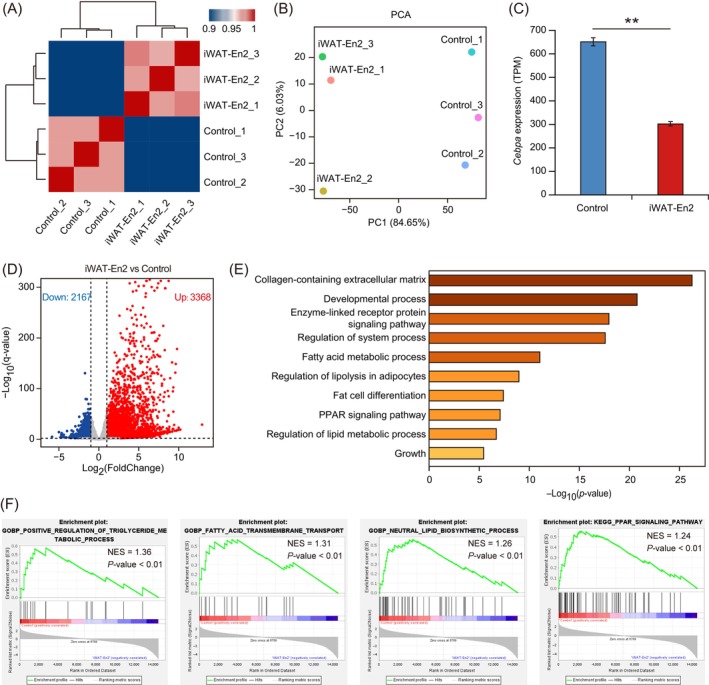
Effects of Cebpa‐L1‐AD‐En2 repression on the transcriptome of adipose tissue. (A) Heatmap showing hierarchical clustering of the Pearson's correlation scores (represented by colour) between the iWAT‐En2 group (*n* = 3) and control group (*n* = 3). (B) Principal component analysis (PCA) plot of normalized RNA‐seq data of iWAT‐En2 group and control group expressed as transcripts per million (TPM). Percentages shown on each axis represent the percentages of variation explained by the principal components. (C) Expression levels (TPM) of *Cebpa* in iWAT of iWAT‐En2 group and control group mice. Bars show the mean and standard deviation (*n* = 3); **p < 0.01. (D) Volcano plot showing differentially expressed genes (DEGs, *q*‐value ≤ 0.01 and |Log_2_(FoldChange)| ≥ 1) between the iWAT‐En2 group and control group. Blue and red dots indicate DEGs up‐regulated in the iWAT‐En2 and control groups, respectively. (E) Functional enrichment analyses of down‐regulated DEGs in iWAT‐En2 group by Metascape. The dot size represents the number of genes, and the colour bar represents the ‐Log_10_(*p*‐value). (F) Gene set enrichment analysis of all expressed genes. A positive value of the normalized enrichment score (NES) indicates enrichment in the control group, and a negative value indicates enrichment in the iWAT‐En2 group.

To examine the effect of Cebpa‐L1‐AD‐En2 repression on adipose tissue development in the transcriptome, we conducted RNA‐seq analysis on the iWAT of iWAT‐En2 group mice and control group mice at 13 weeks of age. Hierarchical clustering showed a high correlation between biological replicates within the iWAT‐En2 and control groups (Figure 8A), while PCA analysis demonstrated a clear separation of samples into two clusters based on their grouping (Figure 8B), indicating distinct differences in transcriptomes between iWAT‐En2 and control groups. RNA‐seq analysis revealed a significant decrease in *Cebpa* expression (*p* < 0.01) in the iWAT of the iWAT‐En2 group compared with the control group (Figure 8C and Table S15). Adipogenesis‐related genes (*Pparg*, *Fabp4*, *Slc2a4*, *Adipoq*, *Lep*, *Lpl*, *Plin1*, and *Pnpla2*) were also significantly down‐regulated (*p* < 0.01) in the iWAT of iWAT‐En2 group (Figure S5D and Table S15). Additionally, we observed no significant alterations in *Pepd* expression between iWAT‐En2 and control groups (Figure S5D and Table S15). Differential expression analysis identified 5535 DEGs (*q*‐value ≤ 0.01 and |Log_2_(FoldChange)| ≥ 1), including 2167 down‐regulated and 3368 up‐regulated genes in the iWAT of iWAT‐En2 group mice (Figure 8D and Table S16). Functional enrichment analysis by Metascape showed that the developmental process, fatty acid metabolic process, regulation of lipolysis in adipocytes, fat cell differentiation, PPAR signalling pathway, and regulation of lipid metabolic process terms were significantly enriched in the control group (Figure 8E). GSEA revealed significant enrichment of positive regulation of triglyceride metabolic process, fatty acid transmembrane transport, lipid biosynthetic process, and PPAR signalling pathway in the control group (Figure 8F). These results indicated that repression of Cebpa‐L1‐AD‐En2 alters the transcriptome of adipose tissue, suppresses adipocyte differentiation and lipid synthesis pathways, and affects adipose tissue development. Overall, our findings demonstrated that Cebpa‐L1‐AD‐En2 is important for adipose tissue development.

## 4 DISCUSSION

Adipocyte differentiation is regulated by a complex cascade of TFs. *Cebpa* is an important TF gene in adipocyte differentiation, playing a vital role in terminal adipocyte differentiation. Precise expression of *Cebpa* is critical for adipocyte differentiation. Although some studies showed that enhancers play an important role in adipocyte differentiation,^42^, ^114^ until now identification of the functional enhancers and mechanisms regulating *Cebpa* expression were largely unknown. In this study, we characterized the chromatin interactomes for *Cebpa* in 3T3‐L1‐PRE and 3T3‐L1‐AD. We identified five active enhancers of *Cebpa* in 3T3‐L1‐AD and further investigated the functions of one of these enhancers, Cebpa‐L1‐AD‐En2, in regulating *Cebpa* expression, adipocyte differentiation, and adipose tissue development.

Changes in gene expression are often accompanied by changes in chromatin interactions.^35^, ^115^ Using the 4C‐seq technique, we studied the genome‐wide chromatin interaction of *Cebpa* during adipogenic differentiation. Our findings revealed that differentiation was associated with more *trans*‐chromatin interactions than *cis*‐chromatin interactions, and an increase in *trans*‐chromatin interactions of *Cebpa*. Considering that *Cebpa* was highly expressed in 3T3‐L1‐AD compared with that in 3T3‐L1‐PRE, and that the regions in which they are located showed higher chromatin activity. We speculate that these active regions might interact with active regions of other chromosomes with a higher interaction frequency than those of low‐active chromatin regions in the genome. Through the integration analysis of 4C‐seq and epigenetic data, we found that 3T3‐L1‐AD had more SISs enriched with active chromatin marks than 3T3‐L1‐PRE, suggesting that more SISs are in an open chromatin state after differentiation, particularly the regions containing the five active enhancers. Histone modification of chromatin can affect gene expression by changing the accessibility of chromatin to TFs.^116^, ^117^ Open chromatin regions can more easily recruit TFs that mediate transcriptional activation of genes through specific DNA sequences. The chromatin of the five active enhancer regions was more open after differentiation, suggesting that they increase the ability to bind TFs and have the potential to regulate *Cebpa* expression. Previous studies have shown that chromatin interactions regulate gene expression by bringing distal regulatory elements in close spatial proximity to gene promoters.^118^, ^119^ Our results showed that four of the five active enhancers exhibited strengthened chromatin interaction after differentiation, especially Cebpa‐L1‐AD‐En2. These enhancers might have increased chromatin interactions with the *Cebpa* promoter in 3 T3‐L1‐AD, possibly activating expression of *Cebpa*. Our findings suggest that the chromatin state and chromatin interaction of these active enhancers are important properties affecting the expression of *Cebpa*.

We investigated the functions of the three enhancers with the highest luciferase reporter activities on *Cebpa* expression during 3T3‐L1 differentiation using an epigenetic modification–CRISPRi system. While repression of Cebpa‐L1‐AD‐En2 and ‐En3 significantly reduced the expression of *Cebpa*, indicating their importance as functional enhancers of *Cebpa*, repression of Cebpa‐L1‐AD‐En7 did not significantly change the expression of *Cebpa*. Reportedly, enhancer redundancy is a remarkably widespread feature of mammalian genomes, and the loss of function of a single enhancer does not necessarily significantly affect gene expression.^120^ Enhancer redundancy not only acts as a regulatory buffer, preventing deleterious phenotypic effects upon loss of an individual enhancer, but also ensures the robustness of gene expression and increases the complexity of gene expression patterns.^120^, ^121^, ^122^ Here, we found that the repression of Cebpa‐L1‐AD‐En7 did not significantly change the expression of *Cebpa*, raising the possibility that Cebpa‐L1‐AD‐En7 may be a redundant enhancer. Our RNA‐seq analysis of dCas9‐KRAB‐En2 and dCas9‐KRAB cells revealed that Cebpa‐L1‐AD‐En2 regulates not only the expression of *Cebpa* but also adipocyte differentiation by affecting the adipogenic differentiation pathway. These results demonstrate the critical role of Cebpa‐L1‐AD‐En2 in transcriptional regulation during adipogenic differentiation. Taken together, our findings suggest that it is possible to manipulate the fate of cell differentiation through the regulation of enhancers, and this may represent a feasible approach for the study of cell fate determination. The luciferase reporter assays revealed the different effects between different active enhancers. Furthermore, to identify enhancers with the strongest regulatory potential on *Cebpa* expression, functional studies were conducted only for the top three enhancers with the highest fluorescence activity, whereas the remaining two enhancers (Cebpa‐L1‐AD‐En1 and ‐En6) were not functionally investigated. Our study evaluated the effects of some active enhancers on *Cebpa* expression and did not fully characterize their respective functional roles. Therefore, it is difficult to fully elucidate the relationship between adipogenic differentiation and *Cebpa* enhancers. Studying the functional mechanisms of each active enhancer will contribute to understanding the coordinated expression of *Cebpa* in the nuclear genome and its comprehensive impact on adipogenic differentiation. Regarding this issue, more in‐depth research remains necessary in the future.

Chromatin loops are an essential feature of eukaryotic genomes and have been broadly accepted as a means for enhancer–promoter communication.^33^, ^123^ Chromatin loop formation via loop extrusion has been widely studied in eukaryotic genomes.^124^, ^125^, ^126^ Chromatin loops can mediate the functional interaction between enhancers and promoters as well as regulate gene expression. Studies have found that a variety of types of chromatin loops mediate enhancer–promoter interactions, such as cohesin‐mediated chromatin loops,^127^, ^128^ CTCF‐mediated chromatin loops,^129^, ^130^ and TFs mediated chromatin loops.^131^, ^132^ In our study, peaks for the SMC1A subunit of cohesin and MED1 were markedly enriched in the functional enhancer Cebpa‐L1‐AD‐En2 and *Cebpa* promoter region, suggesting that cohesin mediates interaction of Cebpa‐L1‐AD‐En2 and the *Cebpa* promoter. The siRNA and 3C‐qPCR experiments further demonstrated that the depletion of cohesin reduced the chromatin interaction between Cebpa‐L1‐AD‐En2 and the *Cebpa* promoter and *Cebpa* expression. These findings indicated that a cohesin‐mediated chromatin loop regulates the functional interaction between Cebpa‐L1‐AD‐En2 and the *Cebpa* promoter. We also found enrichment of a large number of RNA Pol II signals in the Cebpa‐L1‐AD‐En2 region through GRO‐seq and ChIP‐seq data (Figures 2A and 7A). A previous study reported that active enhancers can recruit RNA Pol II and express eRNAs, a new class of non‐coding RNAs that regulate gene expression in a variety of important ways and are correlated with enhancer activity.^133^ For example, eRNAs can establish chromatin accessibility,^134^ stabilize enhancer–promoter looping,^135^, ^136^ regulate the chromatin landscape,^137^ and interact with TFs and chromatin readers.^138^, ^139^, ^140^ Our findings hint that Cebpa‐L1‐AD‐En2 region could be transcribed as an eRNA. Whether the Cebpa‐L1‐AD‐En2 region produces an eRNA and how it might regulate *Cebpa* expression will require further research.

## 5 CONCLUSION

In summary, we have characterized chromatin interactions of *Cebpa* during adipogenic differentiation, identified functional enhancers that regulate *Cebpa* expression, and investigated the role of Cebpa‐L1‐AD‐En2 in regulating adipocyte differentiation and adipose tissue development. Our findings provide insights into the molecular mechanisms of adipocyte differentiation and adipogenesis.

## AUTHOR CONTRIBUTIONS

Mingzhou Li, Keren Long, and Liangpeng Ge conceived and designed the experiments. Xiaokai Li, Sha Zeng, Li Chen, Yu Zhang, Xuemin Li, Biwei Zhang, Duo Su, Qinjiao Du, and Zhining Zhong performed the experiments. Xiaokai Li, Sha Zeng, Jinwei Zhang, Penghao Li, and Anan Jiang involved in data collection and analysis. Xiaokai Li, Sha Zeng, Li Chen, Yu Zhang, Jiaman Zhang, and Haoming Wang conducted high‐throughput data analysis. Xiaokai Li, Sha Zeng, Li Chen, and Yu Zhang drafted the manuscript. Jinwei Zhang, Penghao Li, Anan Jiang, Keren Long, Mingzhou Li and Liangpeng Ge reviewed and edited the article. All authors have read and approved the final article.

## FUNDING INFORMATION

This work was supported by the National Key R & D Program of China (2020YFA0509500 to Mingzhou Li and 2021YFD1301100 to Li Chen), the National Natural Science Foundation of China (U19A2036 and 32225046 to Mingzhou Li, 32102512 to Keren Long, and 32072687 to Liangpeng Ge), the Sichuan Science and Technology Program (2021ZDZX0008, 2022NSFSC0056, and 2022ZHXC0072 to Keren Long, 2021YFYZ0009 to Mingzhou Li), the Major Science and Technology Projects of Tibet Autonomous Region (XZ202101ZD0005N to Mingzhou Li).

## CONFLICT OF INTEREST STATEMENT

The authors declare no conflict of interest.

## Supporting information


**FIGURE S1.** (A) Viewpoint selection and primer design at the *Cebpa* promoter region (−2200 to +500 bp of the transcription start site) for the 4C‐seq experiment. (B) Representative images of 3T3‐L1 preadipocytes (3T3‐L1‐PRE), 3T3‐L1 adipocytes (3T3‐L1‐AD, differentiation for 7 days), and Oil red O staining of 3T3‐L1‐AD. (C) Quantitative real‐time PCR analysis of *Cebpa*, *Pparg*, *Srebf1*, *Adipoq*, *Fabp4*, and *Slc2a4* in 3T3‐L1‐PRE and 3T3‐L1‐AD. Data expressed as mean ± standard deviation (*n* = 3); *p < 0.05, ***p < 0.001. (D) Bar plots showing the percentage of mapped reads in *cis‐*chromosome and *trans*‐chromosome of each 4C data (left). Bar plots showing the percentage of all unique fragment ends at least one mapped read within ±100 kb of the viewpoint (right). (E) Density plot showing the |Log_2_(FoldChange)| distribution for up‐regulated *cis*‐DISs in 3T3‐L1‐AD and 3T3‐L1‐PRE; vertical red‐ and black‐dashed lines indicate the mean values of |Log_2_(FoldChange)| in 3T3‐L1‐AD and 3T3‐L1‐PRE, respectively.

**FIGURE S2.** (A) Manual selection of putative active enhancers of *Cebpa* in 3T3‐L1 preadipocytes (3T3‐L1‐PRE). Integrative Genomics Viewer (IGV) screenshot showing putative active enhancers of *Cebpa* in 3T3‐L1‐PRE. The upper track shows circularized chromosome conformation capture coupled with next‐generation sequencing (4C‐seq) interaction profiles of *Cebpa* in 3T3‐L1‐PRE; 4C signals of replicates are merged. Lower tracks show chromatin immunoprecipitation sequencing (ChIP‐seq) profiles of marks H3K27ac, H3K4me1, H3K4me3, BRD4, p300 and RNA Pol II, and DNAse I hypersensitivity sequencing (DHS‐seq) profiles at *Cebpa* loci in 3T3‐L1‐PRE. The red‐dotted line represents the viewpoint of *Cebpa*. The orange column represents the putative active enhancers. (B) Luciferase reporter assays of the 3T3‐L1 adipocyte‐active enhancers of *Cebpa* in 3T3‐L1 preadipocytes. Luciferase activity was detected 2 days after transfection with the pGL3‐promoter (control) or a pGL3–promoter–enhancer vector. Firefly luciferase signals were normalized with Renilla luciferase signals. Data expressed as mean ± standard deviation of three independent experiments, and *p*‐values were calculated using Student's *t*‐test; *p < 0.05. (C) Chromatin interactions and histone modification (H3K27ac and H3K4me1) of active enhancer regions in 3T3‐L1‐AD and 3T3‐L1‐PRE. The red horizontal lines represent active enhancer regions of 3T3‐L1‐AD. (D) Sequence conservation analysis of Cebpa‐L1‐AD‐En2, ‐En3, ‐En6 and ‐En7 in selected species. UCSC genome browser (http://genome-asia.ucsc.edu/; GRCm38/mm10) was used to assess the sequence conservation. Horizontal red bars indicate conserved elements in 60 vertebrates. Element conservation was measured as logarithm of the odds (LOD) scores of phastCons program elements.

**FIGURE S3.** (A) Proliferating 3T3‐L1 cells expressing dCas9‐KRAB, dCas9‐KRAB‐En2‐sgRNAs, dCas9‐KRAB‐En3‐sgRNAs, or dCas9‐KRAB‐En7‐sgRNAs after 7 days of puromycin selection. (B) Amplification curve of *dCas9* and *β‐actin* (left) and melt curve of *dCas9* and *β‐actin* (right) in dCas9‐KRAB and dCas9‐KRAB‐sgRNAs cells. (C) Gene set enrichment analysis of all expressed genes in dCas9‐KRAB and dCas9‐KRAB‐En2 cells. A positive value of the normalized enrichment score (NES) indicates enrichment in dCas9‐KRAB cells, and a negative value indicates enrichment in dCas9‐KRAB‐En2 cells. (D) Integrative Genomics Viewer screenshot showing *Pepd* expression in 3T3‐L1‐PRE and 3 T3‐L1‐AD by RNA‐seq analysis. The orange column represents the Cebpa‐L1‐AD‐En2 region, and Cebpa‐L1‐AD‐En2 is located in intron 13 of *Pepd*. (E) Amplification curve of *Pepd* and *β‐actin* (left) and melt curve of *Pepd* and *β‐actin* (right) in 3T3‐L1‐PRE and 3T3‐L1‐AD. (F) Relative expression of *Pepd* and *Cebpa* in 3 T3‐L1‐PRE and 3T3‐L1‐AD transfected with either control or *Pepd* siRNA by quantitative real‐time PCR analysis (qRT‐PCR). (G) qRT‐PCR analysis of *Fabp4*, *Lpl*, *Adipoq*, and *Slc2a4* in 3T3‐L1‐AD transfected with either control or *Pepd* siRNA. Data are expressed as mean ± standard deviation (*n* = 3). The *p*‐values were calculated using Student's *t‐*test; *p < 0.05.

**FIGURE S4.** (A) Cebpa‐L1‐AD‐En2 and *Cebpa* are located in the same interaction domain. Alignment of high‐throughput chromosome conformation capture (Hi‐C) data and ChIP‐seq data of CTCF from 3T3‐L1‐AD at the *Cebpa* locus. Upper panel: Hi‐C heatmap showing that Cebpa‐L1‐AD‐En2 and the *Cebpa* promoter are organized in an interaction domain. Lower panel showing ChIP‐seq data of CTCF. The orange column represents the Cebpa‐L1‐AD‐En2, the red column represents the *Cebpa* promoter, and the green column represents the boundaries of the interaction domain. CTCF‐motif position and orientation are indicated by arrows (red arrow: forward core motif, black arrow: reverse core motif). (B) The schematic showing the Hind*III* digestion site and quantitative analysis of chromosome conformation capture (3C‐qPCR) primer location of the *Ercc3* locus. The triangle represents the 3C‐qPCR primer, and the arrow direction represents the primer direction.

**FIGURE S5.** (A) The preliminary experiment of lentiviral local delivery iWAT. The abdomen image after injection of trypan blue into iWAT and skin dissection. The red arrow indicates iWAT. Anterior (towards the head) and posterior (towards the tail). (B) Immunohistological staining assessed the GFP expression of iWAT in control group mice at 9 weeks of age. Paraffin‐fixed WATi sections (5 μm) were stained with GFP antibody (green) and DAPI (blue). Scale bar, 50 μm. (C) Representative haematoxylin and eosin staining image of iWAT of iWAT‐En2 mice and control mice. Scale bars, 100 μm. (D) Heatmap showing the expression levels of genes (*z*‐scores) in iWAT of the iWAT‐En2 and control groups. Genes were subjected to hierarchical clustering.

**TABLE S1.** The primer for qRT‐PCR.

**TABLE S2.** The primer for 4C‐seq library construction.

**TABLE S3.** The detailed information of ChIP‐seq, DHS‐seq, ATAC‐seq, and GRO‐seq data.

**TABLE S4.** The PCR primers of constructed vectors.

**TABLE S5.** CRISPRi single guide RNA (sgRNA) for targeting active enhancers of Cebpa.

**TABLE S6.** Sequences of siRNAs.

**TABLE S7.** The primer for 3C‐qPCR.

**TABLE S8.** The quality metrics of 4C‐seq data.

**TABLE S9.** Genome‐wide chromatin interaction sites of 4C data identified by r3Cseq.

**TABLE S10.** Chromatin interaction sites between replicates identified by r3Cseq.

**TABLE S11.** Differential analysis of interaction sites between 3T3‐L1‐AD and 3T3‐L1‐PRE.

**TABLE S12.** Significant interaction sites overlapping epigenetic features in 3T3‐L1‐PRE and 3T3‐L1‐AD.

**TABLE S13.** Differentially expressed genes between dCas9‐KRAB‐En2 cells and dCas9‐KRAB cells.

**TABLE S14.** Mass spectrometry of Cebpa‐L1‐AD‐En2 pulldown proteins.

**TABLE S15.** Gene expression values (TPM) of inguinal white adipose tissue from control and iWAT‐En2 group mice.

**TABLE S16.** Differentially expressed genes between the inguinal white adipose tissue of control and iWAT‐En2 mice.

## Data Availability

All the sequencing data obtained in this study have been deposited into the NCBI Gene Expression Omnibus (GEO) database under the following accession numbers: 4C‐seq data, GSE222688; mRNA‐seq data, GSE222689 and GSE229849; (https://www.ncbi.nlm.nih.gov/geo/query/acc.cgi?acc=GSE222690, secure token: elsfeecylfwhnyr; https://www.ncbi.nlm.nih.gov/geo/query/acc.cgi?acc=GSE229849, secure token: chmlaaewdzsfjoz). The publically available data used in this study were downloaded from the EBI ENA database (https://www.ebi.ac.uk/ena/browser/home), and the detailed information of public data sets used in this study is listed in the Table S3.
