# Supplementary figures and images for: An intronic enhancer of Cebpa regulates adipocyte differentiation and adipose tissue development via long‐range loop formation
Source: Cell Prolif. 2023 Oct 31;57(3):e13552. doi: 10.1111/cpr.13552 (PMC10905358; doi:10.1111/cpr.13552)

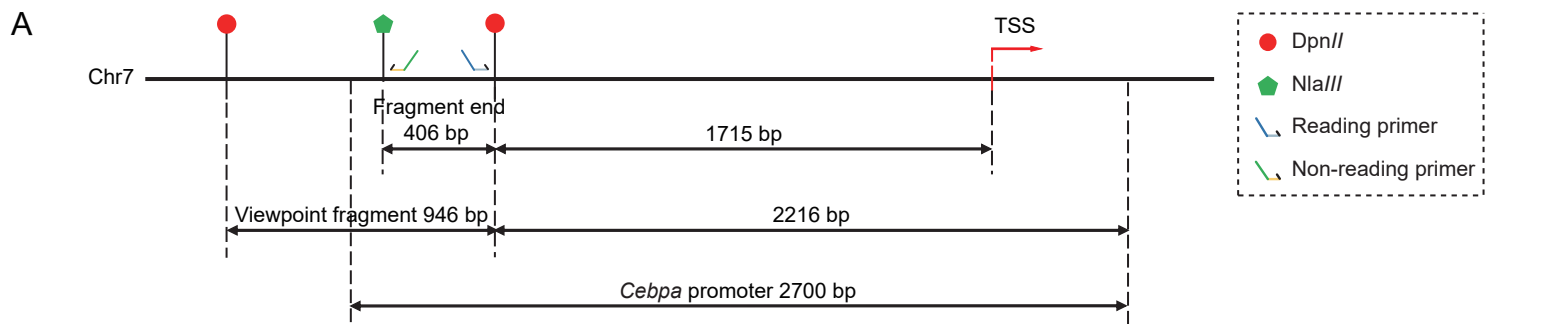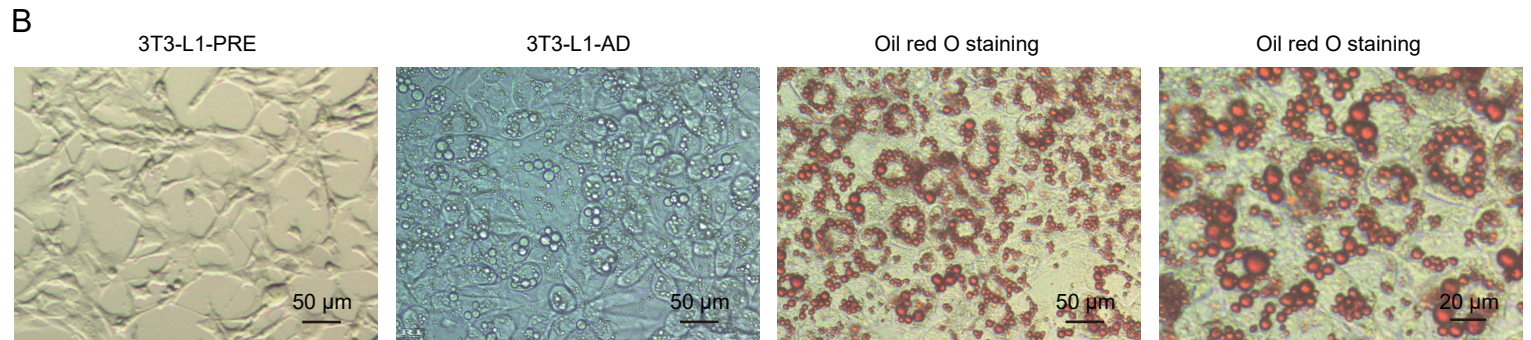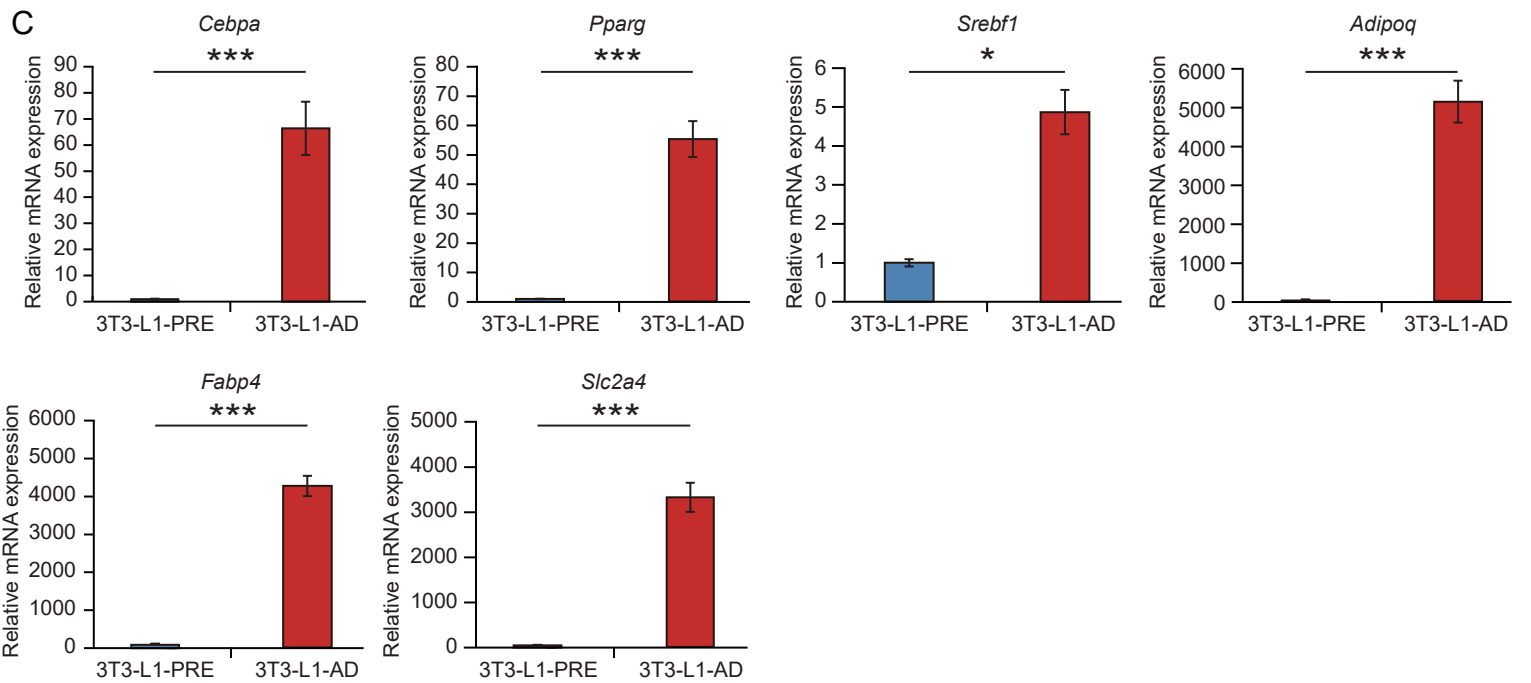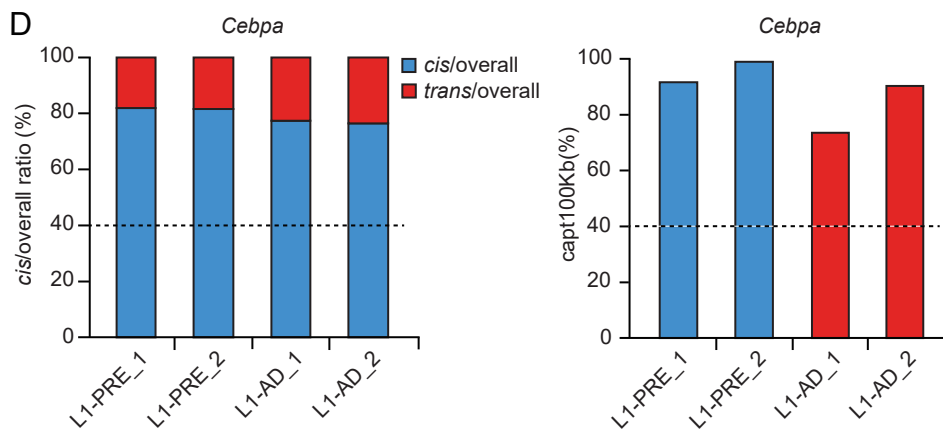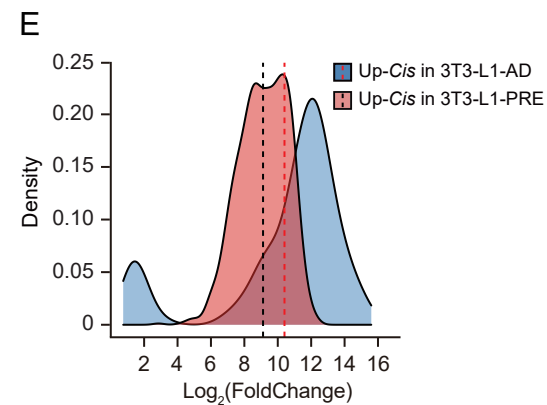

Supplement: Supplementary file 1 — FIGURE S1. (A) Viewpoint selection and primer design at the Cebpa promoter region (−2200 to +500 bp of the transcription start site) for the 4C‐seq experiment. (B) Representative images of 3T3‐L1 preadipocytes (3T3‐L1‐PRE), 3T3‐L1 adipocytes (3T3‐L1‐AD, differentiation for 7 days), and Oil red O staining of 3T3‐L1‐AD. (C) Quantitative real‐time PCR analysis of Cebpa, Pparg, Srebf1, Adipoq, Fabp4, and Slc2a4 in 3T3‐L1‐PRE and 3T3‐L1‐AD. Data expressed as mean ± standard deviation (n = 3); *p < 0.05, ***p < 0.001. (D) Bar plots showing the percentage of mapped reads in cis‐chromosome and trans‐chromosome of each 4C data (left). Bar plots showing the percentage of all unique fragment ends at least one mapped read within ±100 kb of the viewpoint (right). (E) Density plot showing the |Log2(FoldChange)| distribution for up‐regulated cis‐DISs in 3T3‐L1‐AD and 3T3‐L1‐PRE; vertical red‐ and black‐dashed lines indicate the mean values of |Log2(FoldChange)| in 3T3‐L1‐AD and 3T3‐L1‐PRE, respectively. [file CPR-57-e13552-s007.pdf]

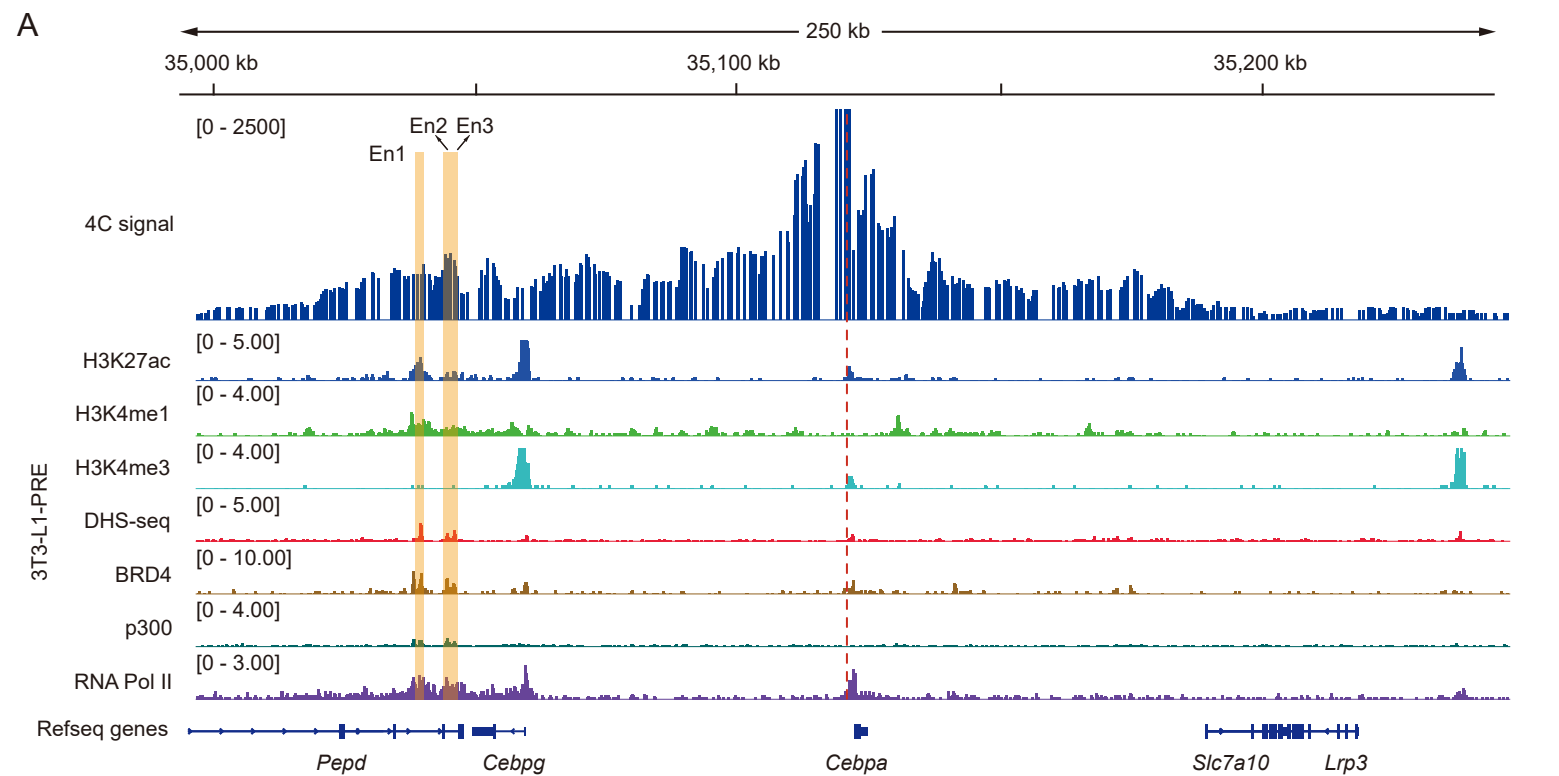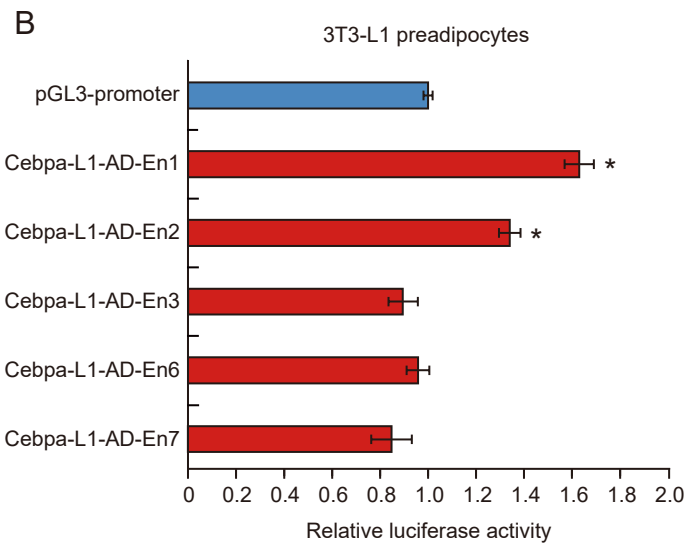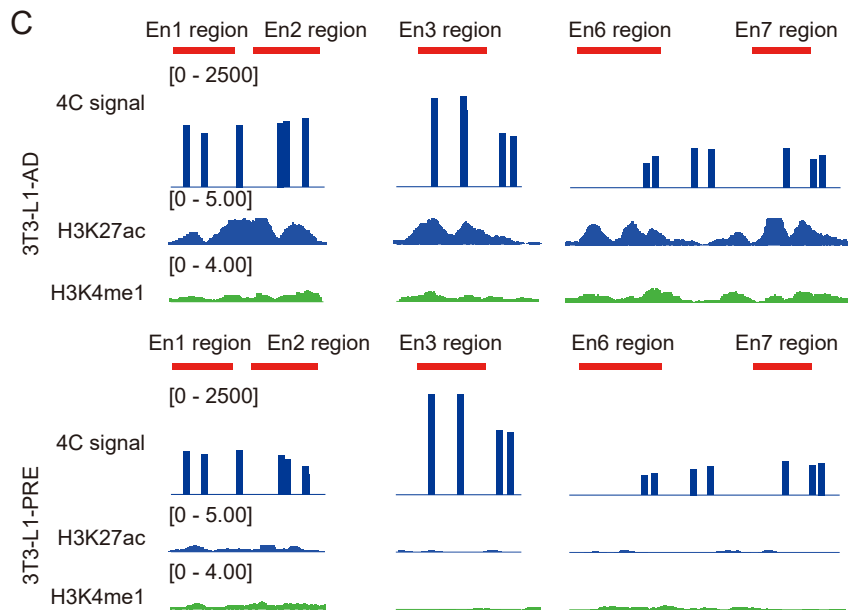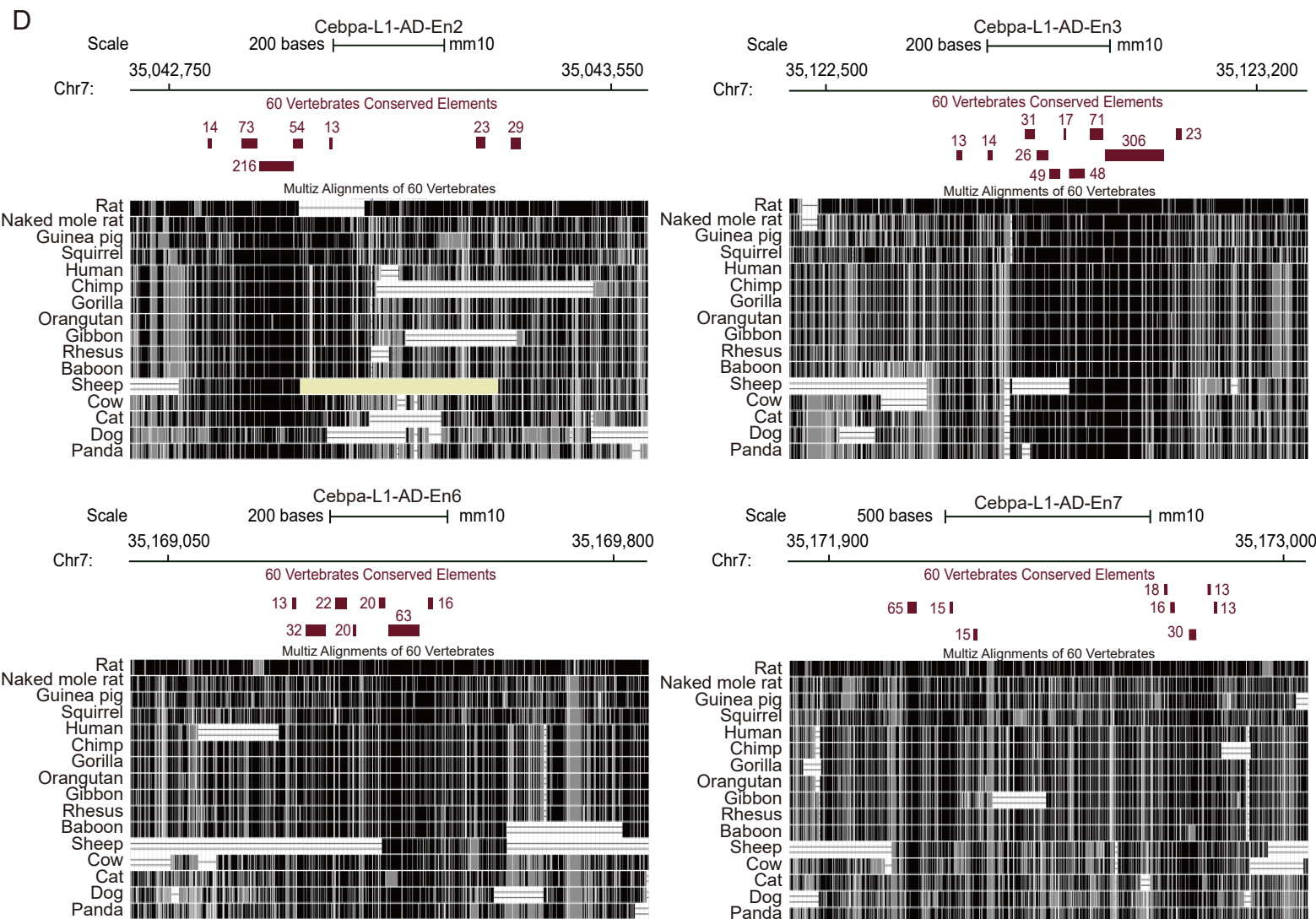

Supplement: Supplementary file 2 — FIGURE S2. (A) Manual selection of putative active enhancers of Cebpa in 3T3‐L1 preadipocytes (3T3‐L1‐PRE). Integrative Genomics Viewer (IGV) screenshot showing putative active enhancers of Cebpa in 3T3‐L1‐PRE. The upper track shows circularized chromosome conformation capture coupled with next‐generation sequencing (4C‐seq) interaction profiles of Cebpa in 3T3‐L1‐PRE; 4C signals of replicates are merged. Lower tracks show chromatin immunoprecipitation sequencing (ChIP‐seq) profiles of marks H3K27ac, H3K4me1, H3K4me3, BRD4, p300 and RNA Pol II, and DNAse I hypersensitivity sequencing (DHS‐seq) profiles at Cebpa loci in 3T3‐L1‐PRE. The red‐dotted line represents the viewpoint of Cebpa. The orange column represents the putative active enhancers. (B) Luciferase reporter assays of the 3T3‐L1 adipocyte‐active enhancers of Cebpa in 3T3‐L1 preadipocytes. Luciferase activity was detected 2 days after transfection with the pGL3‐promoter (control) or a pGL3–promoter–enhancer vector. Firefly luciferase signals were normalized with Renilla luciferase signals. Data expressed as mean ± standard deviation of three independent experiments, and p‐values were calculated using Student's t‐test; *p < 0.05. (C) Chromatin interactions and histone modification (H3K27ac and H3K4me1) of active enhancer regions in 3T3‐L1‐AD and 3T3‐L1‐PRE. The red horizontal lines represent active enhancer regions of 3T3‐L1‐AD. (D) Sequence conservation analysis of Cebpa‐L1‐AD‐En2, ‐En3, ‐En6 and ‐En7 in selected species. UCSC genome browser (http://genome-asia.ucsc.edu/; GRCm38/mm10) was used to assess the sequence conservation. Horizontal red bars indicate conserved elements in 60 vertebrates. Element conservation was measured as logarithm of the odds (LOD) scores of phastCons program elements. [file CPR-57-e13552-s013.pdf]

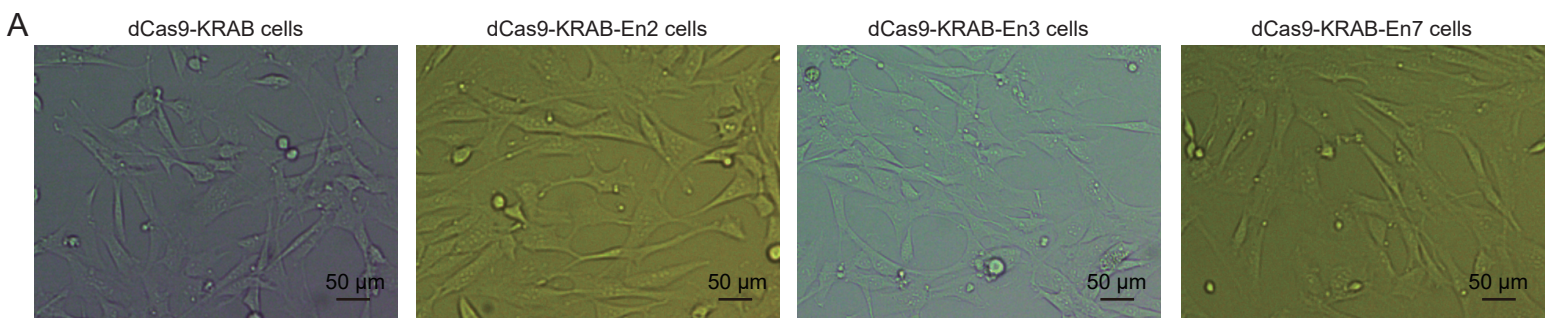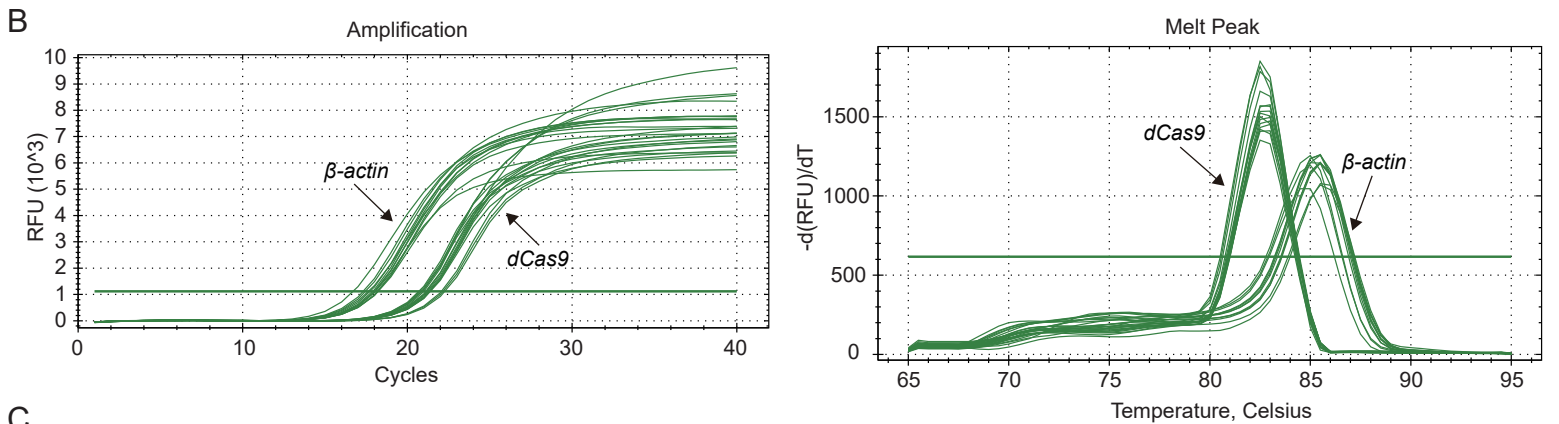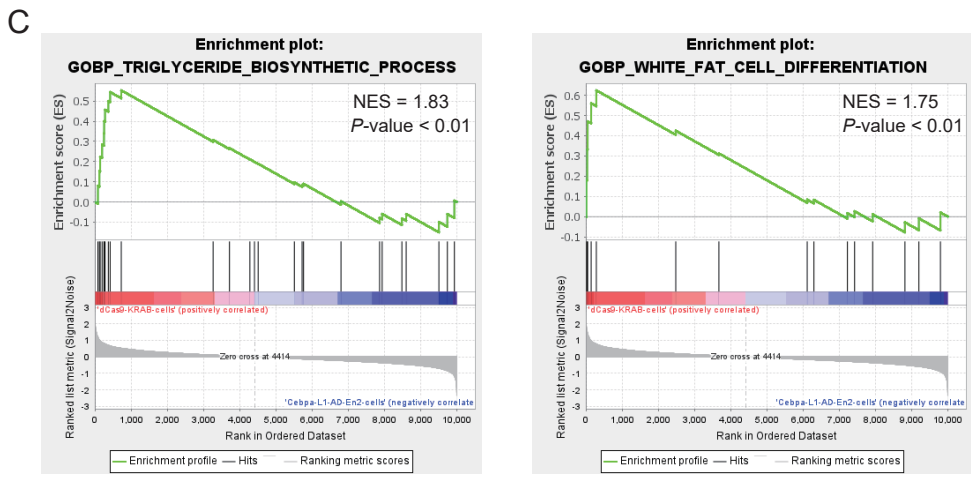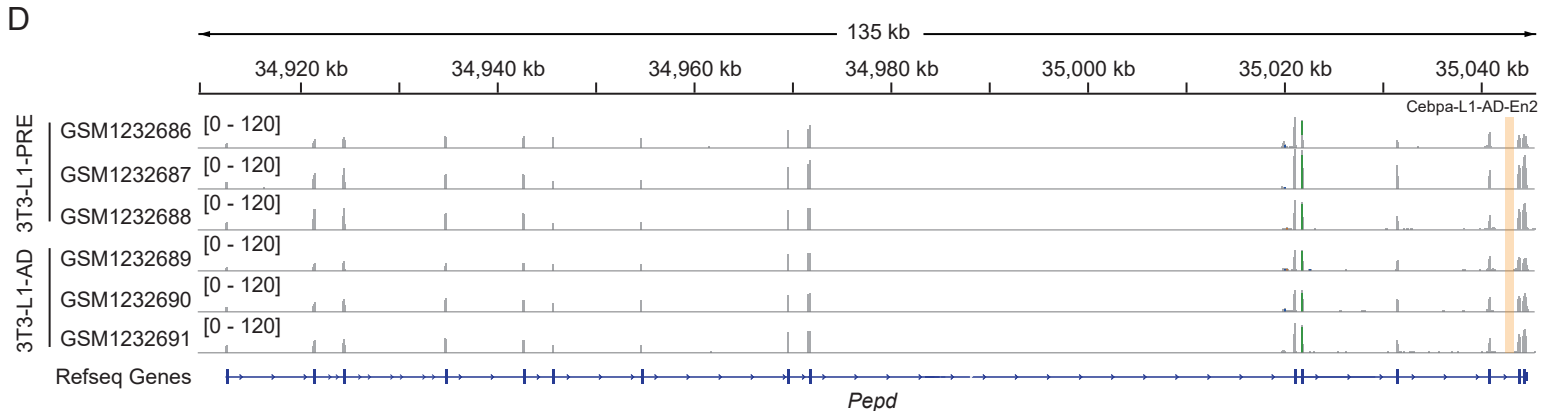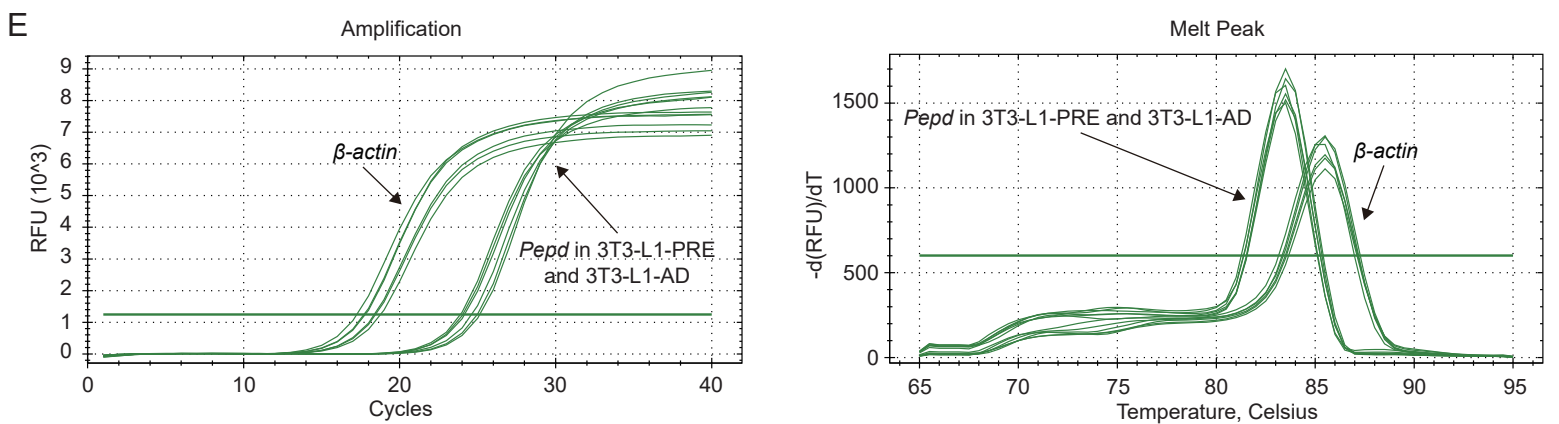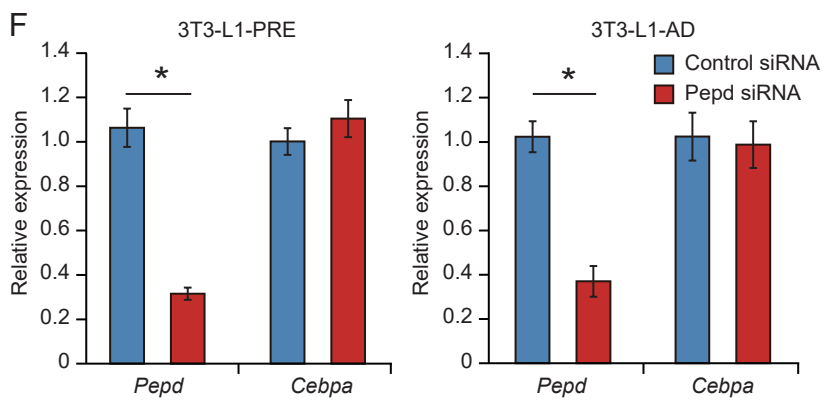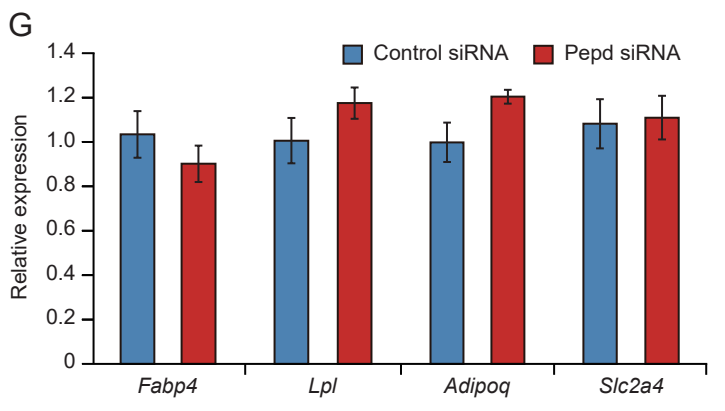

Supplement: Supplementary file 3 — FIGURE S3. (A) Proliferating 3T3‐L1 cells expressing dCas9‐KRAB, dCas9‐KRAB‐En2‐sgRNAs, dCas9‐KRAB‐En3‐sgRNAs, or dCas9‐KRAB‐En7‐sgRNAs after 7 days of puromycin selection. (B) Amplification curve of dCas9 and β‐actin (left) and melt curve of dCas9 and β‐actin (right) in dCas9‐KRAB and dCas9‐KRAB‐sgRNAs cells. (C) Gene set enrichment analysis of all expressed genes in dCas9‐KRAB and dCas9‐KRAB‐En2 cells. A positive value of the normalized enrichment score (NES) indicates enrichment in dCas9‐KRAB cells, and a negative value indicates enrichment in dCas9‐KRAB‐En2 cells. (D) Integrative Genomics Viewer screenshot showing Pepd expression in 3T3‐L1‐PRE and 3 T3‐L1‐AD by RNA‐seq analysis. The orange column represents the Cebpa‐L1‐AD‐En2 region, and Cebpa‐L1‐AD‐En2 is located in intron 13 of Pepd. (E) Amplification curve of Pepd and β‐actin (left) and melt curve of Pepd and β‐actin (right) in 3T3‐L1‐PRE and 3T3‐L1‐AD. (F) Relative expression of Pepd and Cebpa in 3 T3‐L1‐PRE and 3T3‐L1‐AD transfected with either control or Pepd siRNA by quantitative real‐time PCR analysis (qRT‐PCR). (G) qRT‐PCR analysis of Fabp4, Lpl, Adipoq, and Slc2a4 in 3T3‐L1‐AD transfected with either control or Pepd siRNA. Data are expressed as mean ± standard deviation (n = 3). The p‐values were calculated using Student's t‐test; *p < 0.05. [file CPR-57-e13552-s019.pdf]

A

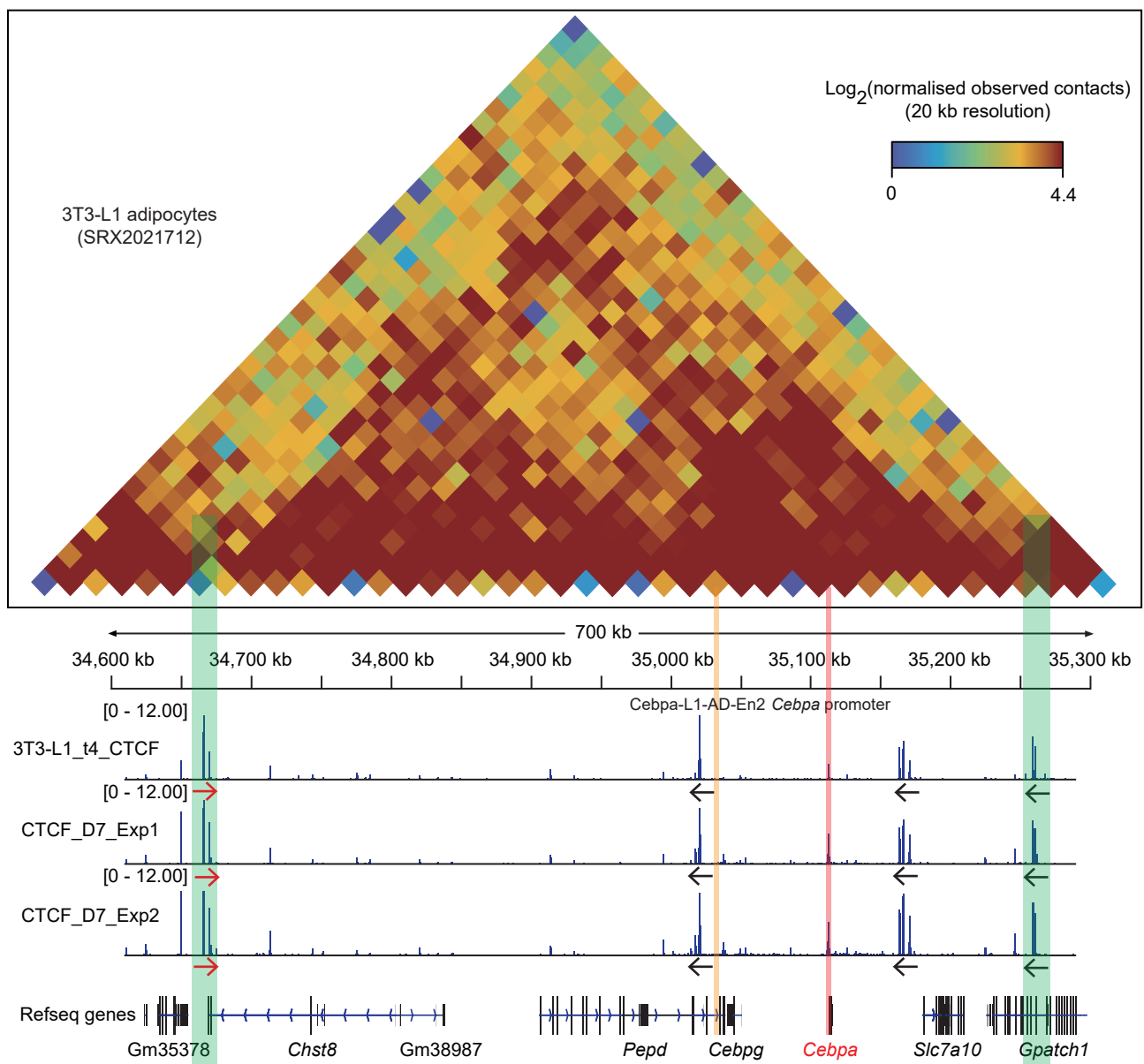

B

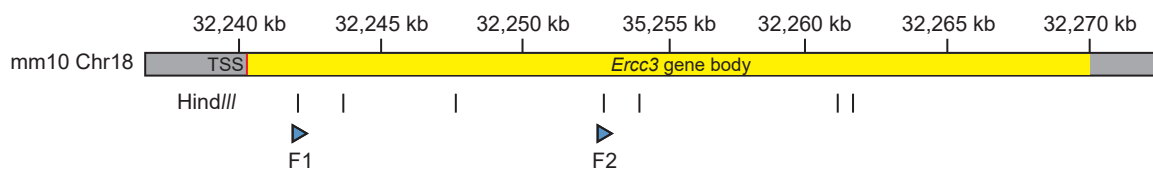

Supplement: Supplementary file 4 — FIGURE S4. (A) Cebpa‐L1‐AD‐En2 and Cebpa are located in the same interaction domain. Alignment of high‐throughput chromosome conformation capture (Hi‐C) data and ChIP‐seq data of CTCF from 3T3‐L1‐AD at the Cebpa locus. Upper panel: Hi‐C heatmap showing that Cebpa‐L1‐AD‐En2 and the Cebpa promoter are organized in an interaction domain. Lower panel showing ChIP‐seq data of CTCF. The orange column represents the Cebpa‐L1‐AD‐En2, the red column represents the Cebpa promoter, and the green column represents the boundaries of the interaction domain. CTCF‐motif position and orientation are indicated by arrows (red arrow: forward core motif, black arrow: reverse core motif). (B) The schematic showing the HindIII digestion site and quantitative analysis of chromosome conformation capture (3C‐qPCR) primer location of the Ercc3 locus. The triangle represents the 3C‐qPCR primer, and the arrow direction represents the primer direction. [file CPR-57-e13552-s014.pdf]

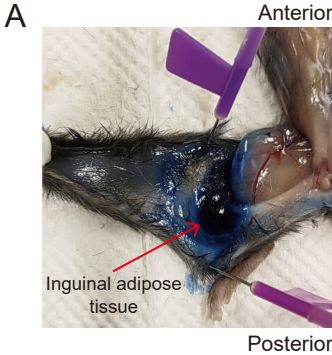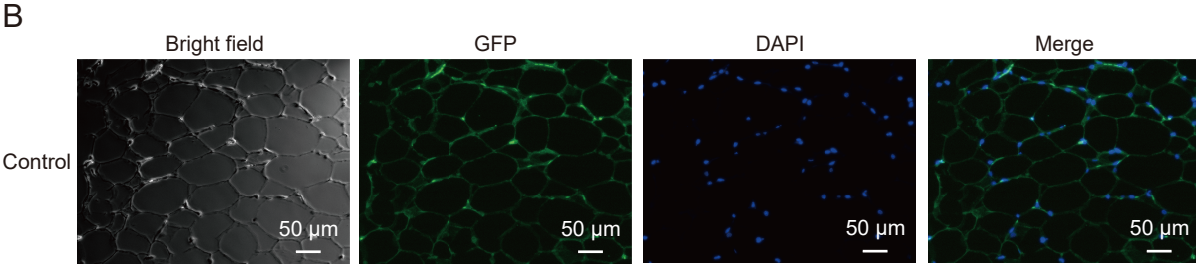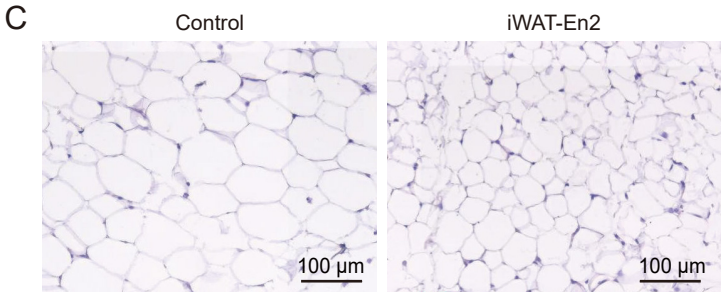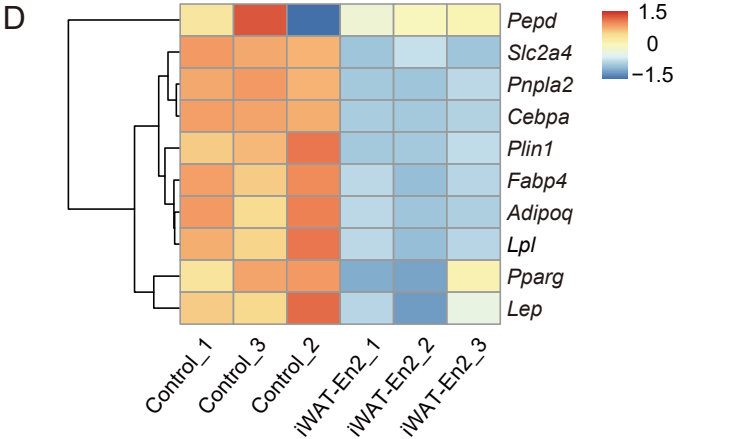

Supplement: Supplementary file 5 — FIGURE S5. (A) The preliminary experiment of lentiviral local delivery iWAT. The abdomen image after injection of trypan blue into iWAT and skin dissection. The red arrow indicates iWAT. Anterior (towards the head) and posterior (towards the tail). (B) Immunohistological staining assessed the GFP expression of iWAT in control group mice at 9 weeks of age. Paraffin‐fixed WATi sections (5 μm) were stained with GFP antibody (green) and DAPI (blue). Scale bar, 50 μm. (C) Representative haematoxylin and eosin staining image of iWAT of iWAT‐En2 mice and control mice. Scale bars, 100 μm. (D) Heatmap showing the expression levels of genes (z‐scores) in iWAT of the iWAT‐En2 and control groups. Genes were subjected to hierarchical clustering. [file CPR-57-e13552-s005.pdf]
